# Supplementary material for: Population-level viremia predicts HIV incidence at the community level across the Universal Testing and Treatment Trials in eastern and southern Africa
Source: PLOS Glob Public Health. 2023 Jul 14;3(7):e0002157. doi: 10.1371/journal.pgph.0002157 (PMC10348573; doi:10.1371/journal.pgph.0002157)
Supplement: S1 Table — (DOCX) [file pgph.0002157.s003.docx]

S1 Table. Additional trial-specific details regarding methodologic approach to estimation of key measures

| Trial | PopART | SEARCH | TasP | Ya Tsie |
| --- | --- | --- | --- | --- |
| **HIV prevalence** | HIV status at baseline was primarily documented within the population cohort using a fourth-generation assay in a laboratory. Estimates were standardized by age and sex to account for non-participation. | The primary source of data for HIV status was rapid HIV antibody testing conducted at the population-level through community health fairs, as well as at home or other locations of choice for persons not attending health fairs.  HIV prevalence estimates were adjusted on age, sex, demographics, and prior testing to account for non-participation.  In each community, an average of all available measures was used to produce midpoint estimates. | TasP combined different data sources to input daily HIV statuses of all residents[1]: rapid HIV antibody testing offered at home, self-reports as HIV-positive, dried blood spots collected at home for research purposes and tested with an Elisa, and medical records from public governmental clinics and trial clinics. Seroconversion date was imputed as well as HIV status prior to a first positive status or after a last negative status. Then, estimates at the midpoint (between trial start and trial closure of each community) were extracted for this analysis, considering only individuals who were active residents at that date [2]. | HIV prevalence was estimated from the population cohort using rapid HIV antibody testing and self-reports. Prevalence was age-sex standardized according to the 2011 Botswana Population Census. |
| **Non-suppression among PLHIV** | HIV RNA testing was performed for all HIV-positive participants of the population cohort at 24 months and only in a subsample of 75 participants per community at 0, 12, and 36 months. | HIV RNA measures were collected at the population-level through community health fairs, as well as at home or other locations of choice for persons not attending health fairs.  Estimates were adjusted for age, sex, demographics, prior testing, prior treatment initiation, and prior suppression to account for potentially differential suppression status among HIV-positive persons for whom viral load measures were not obtained[3].  In each community, an average of all available measures was used to produce midpoint estimates. | HIV RNA data were obtained from trial clinics or routine electronic clinical care records.  Linear interpolation was performed between time points to estimate daily viremia status.  Individuals with undocumented viral load, not on ART or in care in the private sector (and therefore unobserved) at any given time point were considered as having a detectable viremia [1]. | HIV RNA testing was performed on all HIV-positive participants upon identification and again at the final visit. |
| **HIV incidence** | Cumulative HIV incidence was evaluated between months 12 and 36 among individuals from the population cohort aged 18-44 years and HIV-negative at baseline, including individuals newly enrolled in the population cohort at M12 and M24. Incidence estimates were age-sex standardized. | HIV incidence was estimated between months 0 and 36 among individuals HIV-negative at baseline and still resident at endline (excluding in and outmigrants). Estimates used in this paper are not adjusted (in sensitivity analyses, adjustment for characteristics of those measured had minimal impact). | TasP’s primary analysis[4] used only data from dried blood spots collected at home.  In this paper, incidence estimates were refined by taking into account the evolution of residency status, including in-migration and outmigration, as well as complementary data on HIV statuses (rapid HIV tests performed at home, self-reported status, previous clinic records).  These estimates considered varying individual follow-up time (from first to last known HIV status) within the trial area, imputed date of seroconversion (between last negative and first positive status), excluding seroconversions occurring while not residing in the trial area. | Incidence was computed within the survey population cohort among individuals HIV-negative at baseline (therefore excluding immigrants). |

1. Larmarange J, Diallo MH, McGrath N, Iwuji C, Plazy M, Thiébaut R, et al. Temporal trends of population viral suppression in the context of Universal Test and Treat: the ANRS 12249 TasP trial in rural South Africa. J Int AIDS Soc. 2019;22: e25402. doi:10.1002/jia2.25402

2. Larmarange J, Diallo MH, McGrath N, Iwuji C, Plazy M, Thiébaut R, et al. The impact of population dynamics on the population HIV care cascade: results from the ANRS 12249 Treatment as Prevention trial in rural KwaZulu-Natal (South Africa). J Int AIDS Soc. 2018;21: e25128. doi:10.1002/jia2.25128

3. Balzer LB, Ayieko J, Kwarisiima D, Chamie G, Charlebois ED, Schwab J, et al. Far from MCAR: Obtaining Population-level Estimates of HIV Viral Suppression. Epidemiology. 2020;31: 620–627. doi:10.1097/EDE.0000000000001215

4. Iwuji C, Orne-Gliemann J, Larmarange J, Balestre E, Thiebaut R, Tanser F, et al. Universal test and treat and the HIV epidemic in rural South Africa: a phase 4, open-label, community cluster randomised trial. Lancet HIV. 2018;5: e116–e125. doi:10.1016/S2352-3018(17)30205-9
