# Supplementary material for: Population-level viremia predicts HIV incidence at the community level across the Universal Testing and Treatment Trials in eastern and southern Africa
Source: PLOS Glob Public Health. 2023 Jul 14;3(7):e0002157. doi: 10.1371/journal.pgph.0002157 (PMC10348573; doi:10.1371/journal.pgph.0002157)
Supplement: S2 Table — (DOCX) [file pgph.0002157.s004.docx]

S2 Table. Median [minimum – maximum] values of HIV prevalence, prevalence of non-suppression at midpoint, population-level viremia and HIV incidence across communities, per trial

| Trial | PopART | SEARCH | TasP | Ya Tsie | TOTAL |
| --- | --- | --- | --- | --- | --- |
| **Number of communities** | 21 | 32 | 22 | 30 | 105 |
| **HIV prevalence** | 20.4% [3.2% - 32.1%] | 6.6% [2.2% - 21.7%] | 28.1% [17.3% - 41.1%] | 27.1% [15.6% - 39.8%] | 22.2% [2.2% - 41.1%] |
| *- persons included in denominator / trial* | *37 006* | *187 375* | *20 978* | *12 570* | *257 929* |
| *- average persons per cluster* | *1 762* | *5 855* | *953* | *419* | *2 456* |
| **Prevalence of non-suppression** | 34.4% [24.7% - 70.4%] | 41.1% [25.2% - 59.5%] | 61.7% [53.4% - 69.3%] | 12.4% [3.0% - 30.0%] | 34.8% [3.0% -70.4%] |
| *- PLHIV included in the analysis / trial* | *6 233* | *16 209* | *6 617* | *2 318* | *31 377* |
| *- average PLHIV per cluster* | *296.8* | *506.5* | *300.8* | *77.2* | *298.8* |
| **Population-level viremia** | 6.4% [2.3% - 11.4%] | 2.7% [0.6% - 9.5%] | 17.8% [10.6% - 25.2%] | 3.3% [0.8% - 8.2%] | 5.2% [0.6% - 25.2%] |
| **HIV incidence (per 100 PY)** | 1.32 [0.45 - 2.32] | 0.27 [0.03 - 0.60] | 2.11 [1.41 - 3.46] | 0.60 [0.23 - 1.81] | 0.78 [0.03 – 3.46] |
| *- observed person-years / trial* | *39 702* | *270 759* | *26 832* | *8 551* | *345 844* |
| *- average person-years per cluster* | *1 891* | *8 461* | *1 220* | *285* | *3 294* |
