# Supplementary material for: Population-level viremia predicts HIV incidence at the community level across the Universal Testing and Treatment Trials in eastern and southern Africa
Source: PLOS Glob Public Health. 2023 Jul 14;3(7):e0002157. doi: 10.1371/journal.pgph.0002157 (PMC10348573; doi:10.1371/journal.pgph.0002157)
Supplement: S3 Table — Estimates based on community-level linear regressions. (DOCX) [file pgph.0002157.s005.docx]

S3 Table. Relationship between observed population-level viremia and HIV incidence, by trial and trial arm. Estimates based on community-level linear regressions.

|  | Coefficient | [95% CI] | p |
| --- | --- | --- | --- |
| **Slope** (under assumptions, absolute change in expected counterfactual HIV incidence per 100 person-years per hypothetical 10 percentage points absolute change in prevalence of non-suppression) | | | |
| - PopART · control arm | 1.812 | [0.874, 2.749] | <0.001 |
| - PopART · intervention arms | 1.821 | [0.891, 2.751] | <0.001 |
| - SEARCH · control arm | 0.408 | [-0.154, 0.970] | 0.153 |
| - SEARCH · intervention arm | 0.564 | [-0.200, 1.327] | 0.146 |
| - TasP · control arm | 0.461 | [-0.013, 0.936] | 0.057 |
| - TasP · intervention arm | 0.727 | [0.231, 1.223] | 0.005 |
| - Ya Tsie · control arm | -0.370 | [-1.434, 0.694] | 0.491 |
| - Ya Tsie · intervention arm | 0.804 | [-0.397, 2.006] | 0.187 |
| **Intercept** (under assumptions, expected counterfactual HIV incidence per 100 person-years extrapolated to scenario with 0% prevalence of non-suppression) | | | |
| - PopART · control arm | 0.30 | [-0.40, 1.00] | 0.394 |
| - PopART · intervention arms | 0.17 | [-0.41, 0.75] | 0.569 |
| - SEARCH · control arm | 0.10 | [-0.19, 0.40] | 0.478 |
| - SEARCH · intervention arm | 0.09 | [-0.20, 0.38] | 0.552 |
| - TasP · control arm | 1.34 | [0.43, 2.25] | 0.004 |
| - TasP · intervention arm | 0.80 | [-0.09, 1.69] | 0.079 |
| - Ya Tsie · control arm | 1.11 | [0.57, 1.65] | <0.001 |
| - Ya Tsie · intervention arm | 0.37 | [0.02, 0,71] | 0.038 |
