# Supplementary material for: Population-level viremia predicts HIV incidence at the community level across the Universal Testing and Treatment Trials in eastern and southern Africa
Source: PLOS Glob Public Health. 2023 Jul 14;3(7):e0002157. doi: 10.1371/journal.pgph.0002157 (PMC10348573; doi:10.1371/journal.pgph.0002157)
Supplement: S4 Table — Estimates based on community-level linear regressions. (DOCX) [file pgph.0002157.s006.docx]

S4 Table. Relationship between observed population-level viremia and HIV incidence, by trial and country. Estimates based on community-level linear regressions.

|  | Coefficient | [95% CI] | p |
| --- | --- | --- | --- |
| **Slope** (under assumptions, absolute change in expected counterfactual HIV incidence per 100 person-years per hypothetical 10 percentage points absolute change in prevalence of non-suppression) | | | |
| - PopART · South Africa | 2.221 | [1.455, 2.987] | <0.001 |
| - PopART · Zambia | 1.035 | [-0.185, 2.256] | 0.095 |
| - SEARCH · Kenya | 0.332 | [-0.639, 1.304] | 0.499 |
| - SEARCH · Uganda | 0.688 | [-0.715, 2.091] | 0.333 |
| - TasP · South Africa | 0.599 | [0.257, 0.940] | <0.001 |
| - Ya Tsie · Botswana | 0.675 | [0.039, 1.310] | 0.038 |
| **Intercept** (under assumptions, expected counterfactual HIV incidence per 100 person-years extrapolated to scenario with 0% prevalence of non-suppression) | | | |
| - PopART · South Africa | -0.10 | [-0.64, 0.44] | 0.724 |
| - PopART · Zambia | 0.74 | [-0.05, 1.52] | 0.065 |
| - SEARCH · Kenya | 0.18 | [-0.48, 0.84] | 0.589 |
| - SEARCH · Uganda | 0.06 | [-0.27, 0.38] | 0.731 |
| - TasP · South Africa | 1.05 | [0.42, 1.69] | 0.001 |
| - Ya Tsie · Botswana | 0.50 | [0.24, 0.76] | <0.001 |
