# Supplementary material for: Population-level viremia predicts HIV incidence at the community level across the Universal Testing and Treatment Trials in eastern and southern Africa
Source: PLOS Glob Public Health. 2023 Jul 14;3(7):e0002157. doi: 10.1371/journal.pgph.0002157 (PMC10348573; doi:10.1371/journal.pgph.0002157)
Supplement: S1 File — (ZIP) [file pgph.0002157.s007.zip › analysis.html]

Population-level viremia predicts HIV incidence at the community level across the Universal Testing and Treatment Trials in eastern and southern Africa


# Population-level viremia predicts HIV incidence at the community level across the Universal Testing and Treatment Trials in eastern and southern Africa

Joseph Larmarange, Laura Balzer and Maya Petersen for the UT3C consortium
  
2023-06-19

### Contents

- Table S3. Median [minimum - maximum] values of HIV prevalence, prevalence of non-suppression, population-level viremia and HIV incidence, per trial
- Table 3. Linear relationship between population-level viremia and HIV incidence, by trial and gender
- Figure 2. Relationship between population-level viremia and HIV incidence, by trial
- Figure S4. Relationship between population-level viremia and HIV incidence, by trial and arm
- Table S5. Linear relationship between population-level viremia and HIV incidence, by trial and arm
- Figure S6. Relationship between population-level viremia and HIV incidence, by trial and country
- Table S7. Linear relationship between population-level viremia and HIV incidence, by trial and country
- Figure 3. Cross-gendered relationship between population-level viremia and HIV incidence, by trial.
- Table 4. Linear relationship between the prevalence of non-suppression (among PLHIV) and HIV incidence, by trial.
- Figure 4. Relationship between prevalence of non-suppression and HIV incidence, by trial
- Table 5. Estimates of the magnitude of expected incidence reduction due to the observed reduction of viral suppression

```
library(tidyverse)
library(gtsummary)
library(khroma)
library(GGally)
library(scales)
library(hrbrthemes)
library(cowplot)
library(extrafont)
library(labelled)
library(marginaleffects)
```

```
clusters <- read_csv("data.csv")
clusters$intervention <- as.logical(clusters$intervention)
```

## Table S3. Median [minimum - maximum] values of HIV prevalence, prevalence of non-suppression, population-level viremia and HIV incidence, per trial

```
clusters <- clusters %>%
  set_variable_labels(
    study = "Trial",
    arm_study = "Trial & Trial arm",
    trial_country = "Trial & Country",
    prevalence = "HIV prevalence",
    non_suppression = "Prevalence of non-suppression",
    viremia = "Population-level viremia",
    incidence = "HIV incidence (per 100 PY)"
  )

clusters %>%
  tbl_summary(
    include = c(prevalence, non_suppression, viremia, incidence),
    by = study,
    statistic = all_continuous() ~ "{median} [{min} - {max}]",
    digits = list(
      c(prevalence, non_suppression, viremia) ~ scales::label_percent(accuracy = .1),
      incidence ~ scales::label_percent(accuracy = .01, suffix = "")
    )
  ) %>%
  add_overall(last = TRUE)
```

| **Characteristic** | **PopART**, N = 211 | **SEARCH**, N = 321 | **TasP**, N = 221 | **Ya Tsie**, N = 301 | **Overall**, N = 1051 |
| --- | --- | --- | --- | --- | --- |
| HIV prevalence | 20.4% [3.2% - 32.1%] | 6.6% [2.2% - 21.7%] | 28.1% [17.3% - 41.1%] | 27.1% [15.6% - 39.8%] | 22.2% [2.2% - 41.1%] |
| Prevalence of non-suppression | 34.4% [24.7% - 70.4%] | 41.1% [25.2% - 59.5%] | 61.7% [53.4% - 69.3%] | 12.4% [3.0% - 30.0%] | 34.8% [3.0% - 70.4%] |
| Population-level viremia | 6.4% [2.3% - 11.4%] | 2.7% [0.6% - 9.5%] | 17.8% [10.6% - 25.2%] | 3.3% [0.8% - 8.2%] | 5.2% [0.6% - 25.2%] |
| HIV incidence (per 100 PY) | 1.32 [0.45 - 2.32] | 0.27 [0.03 - 0.60] | 2.11 [1.41 - 3.46] | 0.60 [0.23 - 1.81] | 0.78 [0.03 - 3.46] |
|  |  |  |  |  |  |
| --- | --- | --- | --- | --- | --- |
| 1 Median [Range] | | | | | |

## Table 3. Linear relationship between population-level viremia and HIV incidence, by trial and gender

```
overall <- lm(incidence ~ study:viremia + study - 1, data = clusters)

data_cross_gendered <- clusters %>%
  filter(!is.na(incidence_female) & !is.na(incidence_male)) %>%
  mutate(study = fct_drop(study))

fm <- lm(incidence_female ~ study:viremia_male + study - 1, data = data_cross_gendered)
mf <- lm(incidence_male ~ study:viremia_female + study - 1, data = data_cross_gendered)

tbl_merge(
  tbls = list(
    overall %>% 
      tbl_regression(
        estimate_fun = label_number(.0001),
        pvalue_fun = purrr::partial(style_pvalue, digits = 3)
      ),
    fm %>% 
      tbl_regression(
        estimate_fun = label_number(.0001), 
        pvalue_fun = purrr::partial(style_pvalue, digits = 3)
      ),
    mf %>% 
      tbl_regression(
        estimate_fun = label_number(.0001), 
        pvalue_fun = purrr::partial(style_pvalue, digits = 3)
      )
  ),
  tab_spanner = c("**Overall**", "**Women's incidence / Men's viremia**", "**Men's incidence / Women's viremia**")
)
```

| **Characteristic** | **Overall** | | | **Women’s incidence / Men’s viremia** | | | **Men’s incidence / Women’s viremia** | | |
| --- | --- | --- | --- | --- | --- | --- | --- | --- | --- |
|  | **Beta** | **95% CI**1 | **p-value** | **Beta** | **95% CI**1 | **p-value** | **Beta** | **95% CI**1 | **p-value** |
| Trial |  |  |  |  |  |  |  |  |  |
| PopART | 0.0018 | -0.0026, 0.0061 | 0.423 |  |  |  |  |  |  |
| SEARCH | 0.0011 | -0.0010, 0.0031 | 0.297 |  |  |  |  |  |  |
| TasP | 0.0105 | 0.0042, 0.0169 | 0.001 |  |  |  |  |  |  |
| Ya Tsie | 0.0050 | 0.0024, 0.0076 | <0.001 |  |  |  |  |  |  |
| Trial \* Population-level viremia |  |  |  |  |  |  |  |  |  |
| PopART \* Population-level viremia | 0.1877 | 0.1232, 0.2522 | <0.001 |  |  |  |  |  |  |
| SEARCH \* Population-level viremia | 0.0446 | 0.0004, 0.0889 | 0.048 |  |  |  |  |  |  |
| TasP \* Population-level viremia | 0.0599 | 0.0258, 0.0939 | <0.001 |  |  |  |  |  |  |
| Ya Tsie \* Population-level viremia | 0.0675 | 0.0042, 0.1308 | 0.037 |  |  |  |  |  |  |
| study |  |  |  |  |  |  |  |  |  |
| PopART |  |  |  | 0.0100 | 0.0037, 0.0163 | 0.002 | 0.0031 | -0.0022, 0.0083 | 0.247 |
| SEARCH |  |  |  | 0.0010 | -0.0024, 0.0044 | 0.564 | 0.0012 | -0.0014, 0.0037 | 0.375 |
| TasP |  |  |  | 0.0206 | 0.0135, 0.0277 | <0.001 | 0.0063 | -0.0027, 0.0153 | 0.165 |
| study \* viremia\_male |  |  |  |  |  |  |  |  |  |
| PopART \* viremia\_male |  |  |  | 0.1812 | 0.0643, 0.2980 | 0.003 |  |  |  |
| SEARCH \* viremia\_male |  |  |  | 0.0509 | -0.0316, 0.1335 | 0.223 |  |  |  |
| TasP \* viremia\_male |  |  |  | 0.0476 | -0.0009, 0.0961 | 0.054 |  |  |  |
| study \* viremia\_female |  |  |  |  |  |  |  |  |  |
| PopART \* viremia\_female |  |  |  |  |  |  | 0.0719 | 0.0059, 0.1378 | 0.033 |
| SEARCH \* viremia\_female |  |  |  |  |  |  | 0.0393 | -0.0124, 0.0910 | 0.134 |
| TasP \* viremia\_female |  |  |  |  |  |  | 0.0115 | -0.0320, 0.0550 | 0.600 |
|  |  |  |  |  |  |  |  |  |  |
| --- | --- | --- | --- | --- | --- | --- | --- | --- | --- |
| 1 CI = Confidence Interval | | | | | | | | | |

## Figure 2. Relationship between population-level viremia and HIV incidence, by trial

```
# tricks for extending lm lines to zero
lm_zero <- function(formula,data,...){
  mod <- lm(formula, data)
  class(mod) <- c('lm_zero', class(mod))
  mod
}

predictdf.lm_zero <- 
  function(model, xseq, se, level){
    ## here the main code: truncate to x values at the right
    init_range = range(0, model$model$x)
    xseq <- seq(init_range[1], init_range[2], length.out = 10)
    ggplot2:::predictdf.default(model, xseq[-length(xseq)], se, level)
  }

lm_plot <- function(x, y, colour, colour_label = colour, data = clusters, lm = TRUE, lm_se = FALSE) {
  p <- ggplot(data) +
    aes_string(x = x, y = y, colour = colour, fill = colour) +
    geom_point(aes(shape = intervention), size = 2) +
    theme_ipsum(
      axis = TRUE, 
      axis_col = "black",
      axis_title_size = 12,
      axis_title_face = "bold",
      axis_title_just = "mc"
    ) +
    labs(shape = "Arm", fill = colour_label, colour = colour_label) +
    scale_shape_discrete(labels = c("control", "intervention")) +
    theme(
      legend.position = "bottom",
      legend.box = "vertical",
      legend.text = element_text(size = 12),
      legend.title = element_text(size = 12, face = "bold"),
      axis.text.x = element_text(hjust = 0.25)
    ) +
    scale_x_continuous(label = label_percent(1), expand = c(0,0)) +
    scale_y_continuous(label = label_percent(.1, suffix = ""), expand = c(0,0)) +
    expand_limits(
      x = c(0, max(data[[x]], na.rm = TRUE) * 1.025), 
      y = c(0, max(data[[y]], na.rm = TRUE) * 1.025)
    ) +
    scale_color_bright(drop = TRUE) +
    scale_fill_bright(drop = TRUE)
  
  if (lm)
    p <- p +
      geom_smooth(method = "lm_zero", se = FALSE, linetype = "dotted", size = .5) +
      geom_smooth(method = "lm", se = lm_se, alpha = .2, size = 1)
  
  p
}
```

```
fig2 <- lm_plot(x = "viremia", y = "incidence", colour = "study", colour_label = "Trial") +
  xlab("Population-level Viremia: proportion of all adults (HIV+ & HIV-) with non-suppression") +
  ylab("HIV incidence (per 100 person-years)")
fig2
```

## Figure S4. Relationship between population-level viremia and HIV incidence, by trial and arm

```
fig_s4 <- lm_plot(x = "viremia", y = "incidence", colour = "arm_study", colour_label = "Trial & Arm") +
  xlab("Population-level Viremia: proportion of all adults (HIV+ & HIV-) with non-suppression") +
  ylab("HIV incidence (per 100 person-years)") +
  scale_color_brewer(palette = "Paired") +
  scale_fill_brewer(palette = "Paired") +
  guides(
    fill = guide_legend(nrow = 4, byrow = TRUE),
    colour = guide_legend(nrow = 4, byrow = TRUE)
  )
fig_s4
```

## Table S5. Linear relationship between population-level viremia and HIV incidence, by trial and arm

```
mod_s5 <- lm(incidence ~ arm_study:viremia + arm_study - 1, data = clusters)

mod_s5 %>%
  tbl_regression(
    estimate_fun = label_number(.0001),
    pvalue_fun = purrr::partial(style_pvalue, digits = 3)
  )
```

| **Characteristic** | **Beta** | **95% CI**1 | **p-value** |
| --- | --- | --- | --- |
| Trial & Trial arm |  |  |  |
| PopART · control | 0.0030 | -0.0040, 0.0100 | 0.394 |
| PopART · intervention | 0.0017 | -0.0041, 0.0075 | 0.569 |
| SEARCH · control | 0.0010 | -0.0019, 0.0040 | 0.478 |
| SEARCH · intervention | 0.0009 | -0.0020, 0.0038 | 0.552 |
| TasP · control | 0.0134 | 0.0043, 0.0225 | 0.004 |
| TasP · intervention | 0.0080 | -0.0009, 0.0169 | 0.079 |
| Ya Tsie · control | 0.0111 | 0.0057, 0.0165 | <0.001 |
| Ya Tsie · intervention | 0.0037 | 0.0002, 0.0071 | 0.038 |
| Trial & Trial arm \* Population-level viremia |  |  |  |
| PopART · control \* Population-level viremia | 0.1812 | 0.0874, 0.2749 | <0.001 |
| PopART · intervention \* Population-level viremia | 0.1821 | 0.0891, 0.2751 | <0.001 |
| SEARCH · control \* Population-level viremia | 0.0408 | -0.0154, 0.0970 | 0.153 |
| SEARCH · intervention \* Population-level viremia | 0.0564 | -0.0200, 0.1327 | 0.146 |
| TasP · control \* Population-level viremia | 0.0461 | -0.0013, 0.0936 | 0.057 |
| TasP · intervention \* Population-level viremia | 0.0727 | 0.0231, 0.1223 | 0.005 |
| Ya Tsie · control \* Population-level viremia | -0.0370 | -0.1434, 0.0694 | 0.491 |
| Ya Tsie · intervention \* Population-level viremia | 0.0804 | -0.0397, 0.2006 | 0.187 |
|  |  |  |  |
| --- | --- | --- | --- |
| 1 CI = Confidence Interval | | | |

## Figure S6. Relationship between population-level viremia and HIV incidence, by trial and country

```
fig_s6 <- lm_plot(x = "viremia", y = "incidence", colour = "trial_country", colour_label = "Trial & Country") +
  xlab("Population-level Viremia: proportion of all adults (HIV+ & HIV-) with non-suppression") +
  ylab("HIV incidence (per 100 person-years)")
fig_s6
```

## Table S7. Linear relationship between population-level viremia and HIV incidence, by trial and country

```
mod_s7 <- lm(incidence ~ trial_country:viremia + trial_country - 1, data = clusters)

mod_s7 %>%
  tbl_regression(
    estimate_fun = label_number(.0001),
    pvalue_fun = purrr::partial(style_pvalue, digits = 3)
  )
```

| **Characteristic** | **Beta** | **95% CI**1 | **p-value** |
| --- | --- | --- | --- |
| Trial & Country |  |  |  |
| PopART · South Africa | -0.0010 | -0.0064, 0.0044 | 0.724 |
| PopART · Zambia | 0.0074 | -0.0005, 0.0152 | 0.065 |
| SEARCH · Kenya | 0.0018 | -0.0048, 0.0084 | 0.589 |
| SEARCH · Uganda | 0.0006 | -0.0027, 0.0038 | 0.731 |
| TasP · South Africa | 0.0105 | 0.0042, 0.0169 | 0.001 |
| Ya Tsie · Botswana | 0.0050 | 0.0024, 0.0076 | <0.001 |
| Trial & Country \* Population-level viremia |  |  |  |
| PopART · South Africa \* Population-level viremia | 0.2221 | 0.1455, 0.2987 | <0.001 |
| PopART · Zambia \* Population-level viremia | 0.1035 | -0.0185, 0.2256 | 0.095 |
| SEARCH · Kenya \* Population-level viremia | 0.0332 | -0.0639, 0.1304 | 0.499 |
| SEARCH · Uganda \* Population-level viremia | 0.0688 | -0.0715, 0.2091 | 0.333 |
| TasP · South Africa \* Population-level viremia | 0.0599 | 0.0257, 0.0940 | <0.001 |
| Ya Tsie · Botswana \* Population-level viremia | 0.0675 | 0.0039, 0.1310 | 0.038 |
|  |  |  |  |
| --- | --- | --- | --- |
| 1 CI = Confidence Interval | | | |

## Figure 3. Cross-gendered relationship between population-level viremia and HIV incidence, by trial.

```
cross_fm <- 
  lm_plot(
    x = "viremia_female", y = "incidence_male", 
    colour = "study", colour_label = "Trial",
    data = clusters %>% filter(!is.na(viremia_female))
  ) +
  xlab("Population-level Viremia (women)") +
  ylab("HIV incidence (men)") +
  expand_limits(y = 0.048, x = 0.29)

cross_mf <- 
  lm_plot(
    x = "viremia_male", y = "incidence_female", 
    colour = "study", colour_label = "Trial",
    data = clusters %>% filter(!is.na(viremia_male))
  ) +
  xlab("Population-level Viremia (men)") +
  ylab("HIV incidence (women)") +
  expand_limits(y = 0.048, x = 0.29)

up <- plot_grid(
  cross_mf + theme(legend.position = "none"), 
  cross_fm + theme(legend.position = "none"),
  labels = c("a", "b", ""),
  label_size = 11,
  ncol = 2
)

plot_grid(
  up,
  cross_mf %>% get_legend(),
  ncol = 1,
  rel_heights = c(5, 1)
)
```

## Table 4. Linear relationship between the prevalence of non-suppression (among PLHIV) and HIV incidence, by trial.

**Summary of the approach**

```
# Step 1
mod1 <- lm(incidence ~ viremia + study:prevalence + study - 1, data = clusters)

# Step 2
new_data <- clusters %>% 
  expand(
    nesting(id, study, prevalence),
    non_suppression = seq(from = .05, to = .65, by = .03)
  ) %>%
  mutate(viremia = prevalence * non_suppression)
new_data$predicted_incidence <- predict(mod1, newdata = new_data)

# Step 3
mod2 <- lm(predicted_incidence ~ study:non_suppression + study - 1, data = new_data)
```

**Bootstrap version to compute confidence intervals and p-values:**

```
iteration_coef_mod2 <- function(data) {
  # a subsample with replacement
  # (sampling stratified by study)
  data <- data %>%
    dplyr::group_by(study) %>%
    sample_frac(replace = TRUE)
  mod1 <- lm(incidence ~ viremia + study:prevalence + study - 1, data = data)
  new_data <- data %>% 
    ungroup() %>%
    expand(
      nesting(id, study, prevalence),
      non_suppression = seq(from = .05, to = .65, by = .03)
    ) %>%
    mutate(viremia = prevalence * non_suppression)
    new_data$predicted_incidence <- predict(mod1, newdata = new_data)
    mod2 <- lm(predicted_incidence ~ study:non_suppression + study - 1, data = new_data)
    coef(mod2)
}

set.seed(2021) # for replicability

boot_coef <- lapply(1:1000, function(i){iteration_coef_mod2(clusters)}) %>%
  simplify2array %>%
  t()

boot_ci <- matrixStats::colQuantiles(boot_coef, probs = c(0.025, 0.975))
boot_sd <- matrixStats::colSds(boot_coef)

# compute p-values
beta <- coef(mod2)
zval <- beta / boot_sd
boot_p <- 2 * pt(-abs(beta / boot_sd), df = mod2$df.residual)

res <- cbind(coef = beta, boot_ci, p = boot_p)
res <- res %>% as_tibble(rownames = "term")
res$ci <- paste0(
  style_number(res$`2.5%`, digits = 4),
  ", ",
  style_number(res$`97.5%`, digits = 4)
)
```

```
res %>%
  select(term, coef, ci, p) %>%
  gt::gt() %>%
  gt::fmt_number(2, decimals = 4) %>%
  gt::fmt(4, fns = purrr::partial(style_pvalue, digits = 3))
```

| term | coef | ci | p |
| --- | --- | --- | --- |
| studyPopART | 0.0099 | 0.0058, 0.0134 | <0.001 |
| studySEARCH | 0.0006 | -0.0017, 0.0024 | 0.574 |
| studyTasP | 0.0109 | 0.0003, 0.0196 | 0.022 |
| studyYa Tsie | 0.0054 | 0.0034, 0.0074 | <0.001 |
| studyPopART:non\_suppression | 0.0117 | 0.0020, 0.0241 | 0.032 |
| studySEARCH:non\_suppression | 0.0056 | 0.0009, 0.0114 | 0.031 |
| studyTasP:non\_suppression | 0.0170 | 0.0028, 0.0348 | 0.033 |
| studyYa Tsie:non\_suppression | 0.0158 | 0.0025, 0.0327 | 0.033 |

### Expected counterfactual incidence extraploated to scenario where 95-95-95 were achieved

```
compute_95_95_95 <- function(model) {
  marginaleffects::predictions(
    model,
    newdata = datagrid(non_suppression = 1 - .95^3, study = unique)
  ) %>%
    as_tibble() %>%
    select(study, incidence_95_95_95 = estimate)
}

iteration_95_95_95 <- function(i) {
  # a subsample with replacement
  # (sampling stratified by study)
  data <- clusters %>%
    dplyr::group_by(study) %>%
    sample_frac(replace = TRUE)
  mod1 <- lm(incidence ~ viremia + study:prevalence + study - 1, data = data)
  new_data <- data %>% 
    ungroup() %>%
    expand(
      nesting(id, study, prevalence),
      non_suppression = seq(from = .05, to = .65, by = .03)
    ) %>%
    mutate(viremia = prevalence * non_suppression)
    new_data$predicted_incidence <- predict(mod1, newdata = new_data)
    mod2 <- lm(predicted_incidence ~ study:non_suppression + study - 1, data = new_data)
    compute_95_95_95(mod2) %>%
      mutate(iteration = i)
}

set.seed(2021) # for replicability

boot_data_95_95_95 <- 
  1:1000 %>% 
  map_dfr(iteration_95_95_95)
```

```
boot_ci_95_95_95 <- 
  boot_data_95_95_95 %>%
  group_by(study) %>%
  summarise(
    incidence_95_95_95_low = quantile(incidence_95_95_95, probs = .025),
    incidence_95_95_95_high = quantile(incidence_95_95_95, probs = .975),
    .groups = "drop"
  )

res <- compute_95_95_95(mod2) %>%
  left_join(boot_ci_95_95_95, by = "study")

res %>%
  gt::gt() %>%
  gt::fmt_number(decimals = 4)
```

| study | incidence\_95\_95\_95 | incidence\_95\_95\_95\_low | incidence\_95\_95\_95\_high |
| --- | --- | --- | --- |
| PopART | 0.0116 | 0.0089 | 0.0140 |
| SEARCH | 0.0014 | −0.0001 | 0.0025 |
| TasP | 0.0133 | 0.0052 | 0.0200 |
| Ya Tsie | 0.0076 | 0.0063 | 0.0093 |

## Figure 4. Relationship between prevalence of non-suppression and HIV incidence, by trial

```
predict_data <- clusters
predict_data$incidence <- predict(mod2, newdata = clusters)

predict_data2 <- clusters %>%
  group_by(study) %>%
  summarise(non_suppression = range(0, non_suppression))
predict_data2$incidence <- predict(mod2, newdata = predict_data2)


lm_plot(x = "non_suppression", y = "incidence", colour = "study", colour_label = "Trial", lm = FALSE) +
  xlab("Prevalence of non-suppression: proportion of PLHIV with detectable viremia") +
  ylab("HIV incidence (per 100 person-years)") +
  geom_line(data = predict_data2, size = .5, linetype = "dotted") +
  geom_line(data = predict_data, size = 1)
```

## Table 5. Estimates of the magnitude of expected incidence reduction due to the observed reduction of viral suppression

```
non_supression_data <- tribble(
  ~study, ~arm, ~time_point, ~non_suppression,
  "PopART", "A", "baseline", .46,
  "PopART", "A", "endline", .31,
  "PopART", "B", "baseline", .45,
  "PopART", "B", "endline", .31,
  "PopART", "C", "baseline", .49,
  "PopART", "C", "endline", .40,
  "SEARCH", "C", "baseline", .59,
  "SEARCH", "C", "endline", .32,
  "SEARCH", "I", "baseline", .58,
  "SEARCH", "I", "endline", .21,
  "TasP", "C", "baseline", .74,
  "TasP", "C", "endline", .55,
  "TasP", "I", "baseline", .77,
  "TasP", "I", "endline", .54,
  "Ya Tsie", "C", "baseline", .28,
  "Ya Tsie", "C", "endline", .17,
  "Ya Tsie", "I", "baseline", .30,
  "Ya Tsie", "I", "endline", .12,
)

compute_incidence_reduction <- function(model) {
  trends <- non_supression_data
  trends$pred_incidence <- predict(model, newdata = trends)
  trends %>%
    pivot_wider(names_from = "time_point", values_from = c(non_suppression, pred_incidence)) %>%
    mutate(
      incidence_reduction = pred_incidence_endline - pred_incidence_baseline,
      relative_reduction = abs(incidence_reduction) / pred_incidence_baseline
    )
}

compute_incidence_reduction(mod2) %>%
  select(study, arm, incidence_reduction, relative_reduction) %>%
  mutate(
    incidence_reduction = scales::percent(incidence_reduction, accuracy = .01, suffix = ""),
    relative_reduction = scales::percent(relative_reduction, accuracy = .1)
  ) %>%
  gt::gt()
```

| study | arm | incidence\_reduction | relative\_reduction |
| --- | --- | --- | --- |
| PopART | A | -0.18 | 11.5% |
| PopART | B | -0.16 | 10.8% |
| PopART | C | -0.11 | 6.7% |
| SEARCH | C | -0.15 | 39.0% |
| SEARCH | I | -0.21 | 54.3% |
| TasP | C | -0.32 | 13.8% |
| TasP | I | -0.39 | 16.3% |
| Ya Tsie | C | -0.17 | 17.7% |
| Ya Tsie | I | -0.28 | 28.1% |

**Bootstrap version to compute 95% confidence intervals:**

```
iteration_incidence_reduction <- function(i) {
  # a subsample with replacement
  # (sampling stratified by study)
  data <- clusters %>%
    dplyr::group_by(study) %>%
    sample_frac(replace = TRUE)
  mod1 <- lm(incidence ~ viremia + study:prevalence + study - 1, data = data)
  new_data <- data %>% 
    ungroup() %>%
    expand(
      nesting(id, study, prevalence),
      non_suppression = seq(from = .05, to = .65, by = .03)
    ) %>%
    mutate(viremia = prevalence * non_suppression)
    new_data$predicted_incidence <- predict(mod1, newdata = new_data)
    mod2 <- lm(predicted_incidence ~ study:non_suppression + study - 1, data = new_data)
    compute_incidence_reduction(mod2) %>%
      mutate(iteration = i)
}

set.seed(2021) # for replicability

boot_data <- 
  1:1000 %>% 
  map_dfr(iteration_incidence_reduction)
```

```
boot_ci <- 
  boot_data %>%
  group_by(study, arm) %>%
  summarise(
    incidence_reduction_low = quantile(incidence_reduction, probs = .025),
    incidence_reduction_high = quantile(incidence_reduction, probs = .975),
    relative_reduction_low = quantile(relative_reduction, probs = .025),
    relative_reduction_high = quantile(relative_reduction, probs = .975),
    .groups = "drop"
  )

res <- compute_incidence_reduction(mod2) %>%
  left_join(boot_ci, by = c("study", "arm"))

res %>%
  mutate(
    incidence_reduction = paste0(
      scales::percent(incidence_reduction, accuracy = .01, suffix = ""),
      " [",
      scales::percent(incidence_reduction_low, accuracy = .01, suffix = ""),
      ", ",
      scales::percent(incidence_reduction_high, accuracy = .01, suffix = ""),
      "]"
    ),
    relative_reduction = paste0(
      scales::percent(relative_reduction, accuracy = .1),
      " [",
      scales::percent(relative_reduction_low, accuracy = .1, suffix = ""),
      ", ",
      scales::percent(relative_reduction_high, accuracy = .1, suffix = ""),
      "]"
    )
  ) %>%
  select(study, arm, incidence_reduction, relative_reduction) %>%
  gt::gt()
```

| study | arm | incidence\_reduction | relative\_reduction |
| --- | --- | --- | --- |
| PopART | A | -0.18 [-0.36, -0.03] | 11.5% [2.2, 21.2] |
| PopART | B | -0.16 [-0.34, -0.03] | 10.8% [2.1, 20.1] |
| PopART | C | -0.11 [-0.22, -0.02] | 6.7% [1.3, 12.2] |
| SEARCH | C | -0.15 [-0.31, -0.02] | 39.0% [8.5, 61.9] |
| SEARCH | I | -0.21 [-0.42, -0.03] | 54.3% [11.7, 86.9] |
| TasP | C | -0.32 [-0.66, -0.05] | 13.8% [2.5, 25.3] |
| TasP | I | -0.39 [-0.80, -0.06] | 16.3% [3.1, 29.5] |
| Ya Tsie | C | -0.17 [-0.36, -0.03] | 17.7% [3.8, 27.8] |
| Ya Tsie | I | -0.28 [-0.59, -0.05] | 28.1% [6.1, 43.2] |
