# Supplementary material for: Population-level viremia predicts HIV incidence at the community level across the Universal Testing and Treatment Trials in eastern and southern Africa
Source: PLOS Glob Public Health. 2023 Jul 14;3(7):e0002157. doi: 10.1371/journal.pgph.0002157 (PMC10348573; doi:10.1371/journal.pgph.0002157)
Supplement: S1 File — (ZIP) [file pgph.0002157.s007.zip › analysis.pdf]

Population-level viremia predicts HIV incidence at the community level across the Universal Testing and Treatment Trials in eastern and southern Africa

true

2023-06-19

Contents

Table S3. Median [minimum - maximum] values of HIV prevalence, prevalence of non-suppression, population-level viremia and HIV incidence, per trial . . . 1
Table 3. Linear relationship between population-level viremia and HIV incidence, by trial and gender 2
Figure 2. Relationship between population-level viremia and HIV incidence, by trial . . . 4
Figure S4. Relationship between population-level viremia and HIV incidence, by trial and arm . . 6
Table S5. Linear relationship between population-level viremia and HIV incidence, by trial and arm 7
Figure S6. Relationship between population-level viremia and HIV incidence, by trial and country 7
Table S7. Linear relationship between population-level viremia and HIV incidence, by trial and country . . . 8
Figure 3. Cross-gendered relationship between population-level viremia and HIV incidence, by trial. 9
Table 4. Linear relationship between the prevalence of non-suppression (among PLHIV) and HIV incidence, by trial. . . . 10
Figure 4. Relationship between prevalence of non-suppression and HIV incidence, by trial 13
Table 5. Estimates of the magnitude of expected incidence reduction due to the observed reduction of viral suppression . . . 14

```
library(tidyverse)
library(gtsummary)
library(khroma)
library(GGally)
library(scales)
library(hrbrthemes)
library(cowplot)
library(extrafont)
library(labelled)
library(marginaleffects)
```

```
clusters <- read_csv("data.csv")
clusters$intervention <- as.logical(clusters$intervention)
```

Table S3. Median [minimum - maximum] values of HIV prevalence, prevalence of non-suppression, population-level viremia and HIV incidence, per trial

```
clusters <- clusters %>%
  set_variable_labels(
    study = "Trial",
```

```
    arm_study = "Trial & Trial arm",
    trial_country = "Trial & Country",
    prevalence = "HIV prevalence",
    non_suppression = "Prevalence of non-suppression",
    viremia = "Population-level viremia",
    incidence = "HIV incidence (per 100 PY)"
  )

clusters %>%
  tbl_summary(
    include = c(prevalence, non_suppression, viremia, incidence),
    by = study,
    statistic = all_continuous() ~ "{median} [{min} - {max}]",
    digits = list(
      c(prevalence, non_suppression, viremia) ~ scales::label_percent(accuracy = .1),
      incidence ~ scales::label_percent(accuracy = .01, suffix = "")
    )
  ) %>%
  add_overall(last = TRUE)
```

**Characteristic**	**PopART**, N = 21	**SEARCH**, N = 32	**TasP**, N = 22	**Ya Tsie**, N = 30	**Overall**, N = 105
HIV prevalence	20.4% [3.2% - 32.1%]	6.6% [2.2% - 21.7%]	28.1% [17.3% - 41.1%]	27.1% [15.6% - 39.8%]	22.2% [2.2% - 41.1%]
Prevalence of non-suppression	34.4% [24.7% - 70.4%]	41.1% [25.2% - 59.5%]	61.7% [53.4% - 69.3%]	12.4% [3.0% - 30.0%]	34.8% [3.0% - 70.4%]
Population-level viremia	6.4% [2.3% - 11.4%]	2.7% [0.6% - 9.5%]	17.8% [10.6% - 25.2%]	3.3% [0.8% - 8.2%]	5.2% [0.6% - 25.2%]
HIV incidence (per 100 PY)	1.32 [0.45 - 2.32]	0.27 [0.03 - 0.60]	2.11 [1.41 - 3.46]	0.60 [0.23 - 1.81]	0.78 [0.03 - 3.46]

Table 3. Linear relationship between population-level viremia and HIV incidence, by trial and gender

```
overall <- lm(incidence ~ study:viremia + study - 1, data = clusters)

data_cross_gendered <- clusters %>%
  filter(!is.na(incidence_female) & !is.na(incidence_male)) %>%
  mutate(study = fct_drop(study))

fm <- lm(incidence_female ~ study:viremia_male + study - 1, data = data_cross_gendered)
mf <- lm(incidence_male ~ study:viremia_female + study - 1, data = data_cross_gendered)

tbl_merge(
  tbls = list(
    overall %>%
      tbl_regression(
        estimate_fun = label_number(.0001),
        pvalue_fun = purrr::partial(style_pvalue, digits = 3)
      ),
    fm %>%
      tbl_regression(
```

```
        estimate_fun = label_number(.0001),
        pvalue_fun = purrr::partial(style_pvalue, digits = 3)
      ),
    mf %>%
      tbl_regression(
        estimate_fun = label_number(.0001),
        pvalue_fun = purrr::partial(style_pvalue, digits = 3)
      )
  ),
  tab_spanner = c("**Overall**", "**Women's incidence / Men's viremia**", "**Men's incidence / Women's v
)
```

Characteristic	Beta	95% CI	p-value	Beta	95% CI	p-value	Beta	95% CI	p-value
Trial									
PopART	0.0018	-0.0026, 0.0061	0.423						
SEARCH	0.0011	-0.0010, 0.0031	0.297						
TasP	0.0105	0.0042, 0.0169	0.001						
Ya Tsie	0.0050	0.0024, 0.0076	<0.001						
Trial * Population-level viremia									
PopART * Population-level viremia	0.1877	0.1232, 0.2522	<0.001						
SEARCH * Population-level viremia	0.0446	0.0004, 0.0889	0.048						
TasP * Population-level viremia	0.0599	0.0258, 0.0939	<0.001						
Ya Tsie * Population-level viremia	0.0675	0.0042, 0.1308	0.037						
study									
PopART				0.0100	0.0037, 0.0163	0.002	0.0031	-0.0022, 0.0083	0.247
SEARCH				0.0010	-0.0024, 0.0044	0.564	0.0012	-0.0014, 0.0037	0.375
TasP				0.0206	0.0135, 0.0277	<0.001	0.0063	-0.0027, 0.0153	0.165
study * viremia_male									
PopART * viremia_male				0.1812	0.0643, 0.2980	0.003			
SEARCH * viremia_male				0.0509	-0.0316, 0.1335	0.223			
TasP * viremia_male				0.0476	-0.0009, 0.0961	0.054			

Characteristic	Beta	95% CI	p-value	Beta	95% CI	p-value	Beta	95% CI	p-value
study * viremia_female									
PopART * viremia_female							0.0719	0.0059, 0.1378	0.033
SEARCH * viremia_female							0.0393	-0.0124, 0.0910	0.134
TasP * viremia_female							0.0115	-0.0320, 0.0550	0.600

Figure 2. Relationship between population-level viremia and HIV incidence, by trial

```r
# tricks for extending lm lines to zero
lm_zero <- function(formula,data,...){
  mod <- lm(formula, data)
  class(mod) <- c('lm_zero', class(mod))
  mod
}

predictdf.lm_zero <-
  function(model, xseq, se, level){
    ## here the main code: truncate to x values at the right
    init_range = range(0, model$model$x)
    xseq <- seq(init_range[1], init_range[2], length.out = 10)
    ggplot2:::predictdf.default(model, xseq[-length(xseq)], se, level)
  }

lm_plot <- function(x, y, colour, colour_label = colour, data = clusters, lm = TRUE, lm_se = FALSE) {
  p <- ggplot(data) +
    aes_string(x = x, y = y, colour = colour, fill = colour) +
    geom_point(aes(shape = intervention), size = 2) +
    theme_ipsum(
      axis = TRUE,
      axis_col = "black",
      axis_title_size = 12,
      axis_title_face = "bold",
      axis_title_just = "mc"
    ) +
    labs(shape = "Arm", fill = colour_label, colour = colour_label) +
    scale_shape_discrete(labels = c("control", "intervention")) +
    theme(
      legend.position = "bottom",
      legend.box = "vertical",
      legend.text = element_text(size = 12),
      legend.title = element_text(size = 12, face = "bold"),
      axis.text.x = element_text(hjust = 0.25)
    ) +
    scale_x_continuous(label = label_percent(1), expand = c(0,0)) +
    scale_y_continuous(label = label_percent(.1, suffix = ""), expand = c(0,0)) +
    expand_limits(
```

```
      x = c(0, max(data[[x]], na.rm = TRUE) * 1.025),
      y = c(0, max(data[[y]], na.rm = TRUE) * 1.025)
    ) +
    scale_color_bright(drop = TRUE) +
    scale_fill_bright(drop = TRUE)

  if (lm)
    p <- p +
      geom_smooth(method = "lm_zero", se = FALSE, linetype = "dotted", size = .5) +
      geom_smooth(method = "lm", se = lm_se, alpha = .2, size = 1)

  p
}
```

```
fig2 <- lm_plot(x = "viremia", y = "incidence", colour = "study", colour_label = "Trial") +
  xlab("Population-level Viremia: proportion of all adults (HIV+ & HIV-) with non-suppression") +
  ylab("HIV incidence (per 100 person-years)")
fig2
```

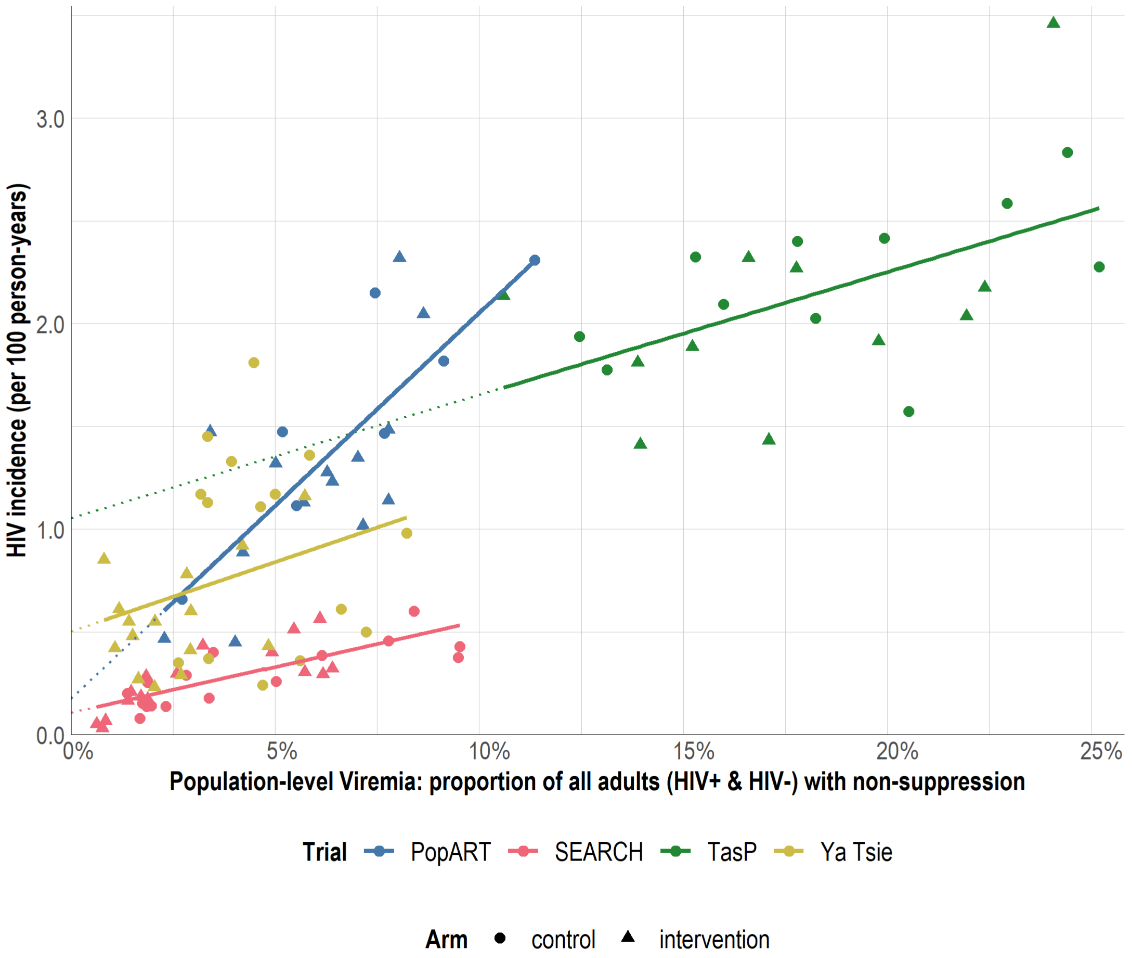

Figure S4. Relationship between population-level viremia and HIV incidence, by trial and arm

```
fig_s4 <- lm_plot(x = "viremia", y = "incidence", colour = "arm_study", colour_label = "Trial & Arm") +
  xlab("Population-level Viremia: proportion of all adults (HIV+ & HIV-) with non-suppression") +
  ylab("HIV incidence (per 100 person-years)") +
  scale_color_brewer(palette = "Paired") +
  scale_fill_brewer(palette = "Paired") +
  guides(
    fill = guide_legend(nrow = 4, byrow = TRUE),
    colour = guide_legend(nrow = 4, byrow = TRUE)
  )
fig_s4
```

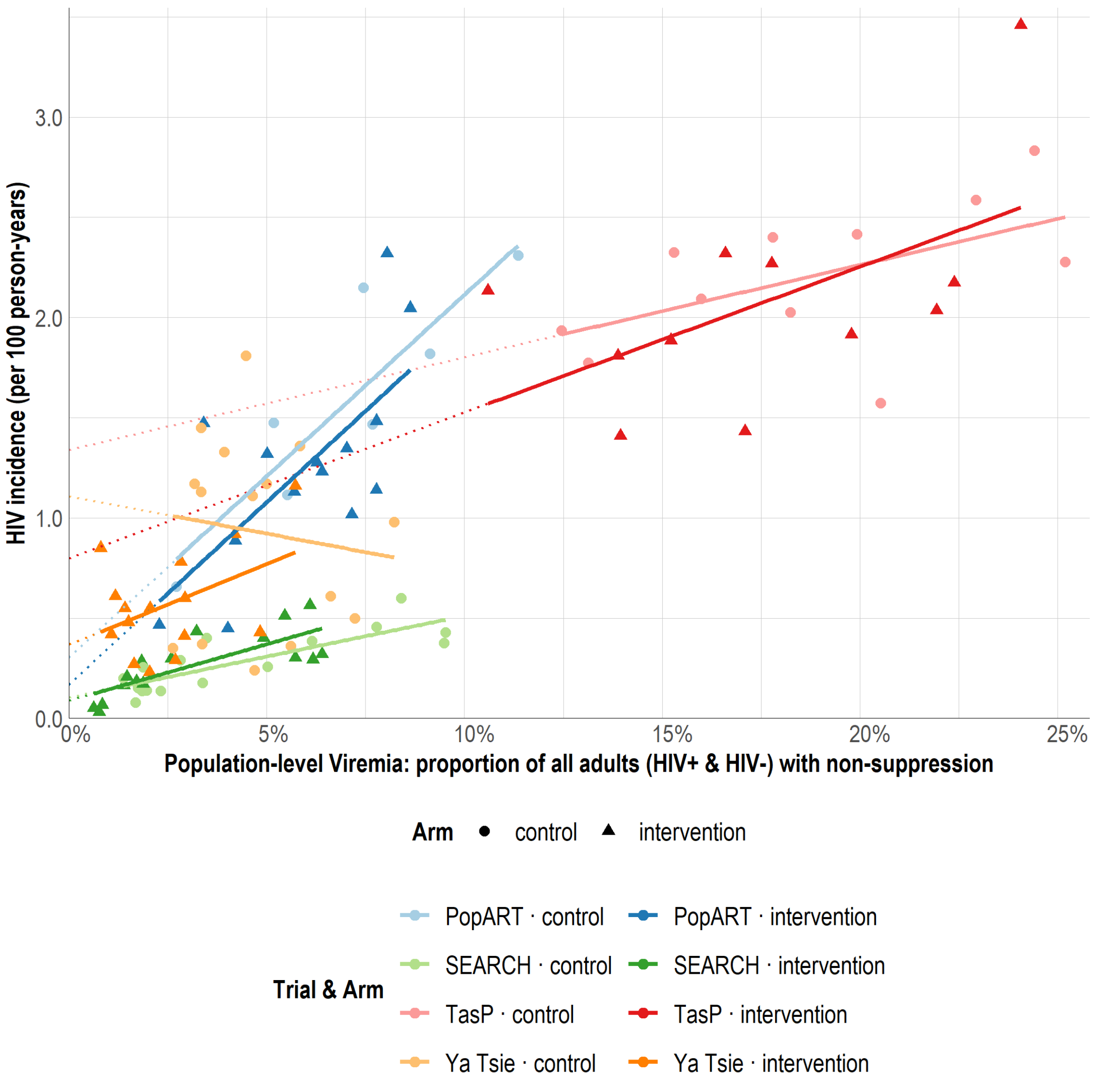

Table S5. Linear relationship between population-level viremia and HIV incidence, by trial and arm

```
mod_s5 <- lm(incidence ~ arm_study:viremia + arm_study - 1, data = clusters)

mod_s5 %>%
  tbl_regression(
    estimate_fun = label_number(.0001),
    pvalue_fun = purrr::partial(style_pvalue, digits = 3)
  )
```

Characteristic	Beta	95% CI	p-value
Trial & Trial arm			
PopART · control	0.0030	-0.0040, 0.0100	0.394
PopART · intervention	0.0017	-0.0041, 0.0075	0.569
SEARCH · control	0.0010	-0.0019, 0.0040	0.478
SEARCH · intervention	0.0009	-0.0020, 0.0038	0.552
TasP · control	0.0134	0.0043, 0.0225	0.004
TasP · intervention	0.0080	-0.0009, 0.0169	0.079
Ya Tsie · control	0.0111	0.0057, 0.0165	<0.001
Ya Tsie · intervention	0.0037	0.0002, 0.0071	0.038
Trial & Trial arm * Population-level viremia			
PopART · control * Population-level viremia	0.1812	0.0874, 0.2749	<0.001
PopART · intervention * Population-level viremia	0.1821	0.0891, 0.2751	<0.001
SEARCH · control * Population-level viremia	0.0408	-0.0154, 0.0970	0.153
SEARCH · intervention * Population-level viremia	0.0564	-0.0200, 0.1327	0.146
TasP · control * Population-level viremia	0.0461	-0.0013, 0.0936	0.057
TasP · intervention * Population-level viremia	0.0727	0.0231, 0.1223	0.005
Ya Tsie · control * Population-level viremia	-0.0370	-0.1434, 0.0694	0.491
Ya Tsie · intervention * Population-level viremia	0.0804	-0.0397, 0.2006	0.187

Figure S6. Relationship between population-level viremia and HIV incidence, by trial and country

```
fig_s6 <- lm_plot(x = "viremia", y = "incidence", colour = "trial_country", colour_label = "Trial & Cou
  xlab("Population-level Viremia: proportion of all adults (HIV+ & HIV-) with non-suppression") +
  ylab("HIV incidence (per 100 person-years)")
fig_s6
```

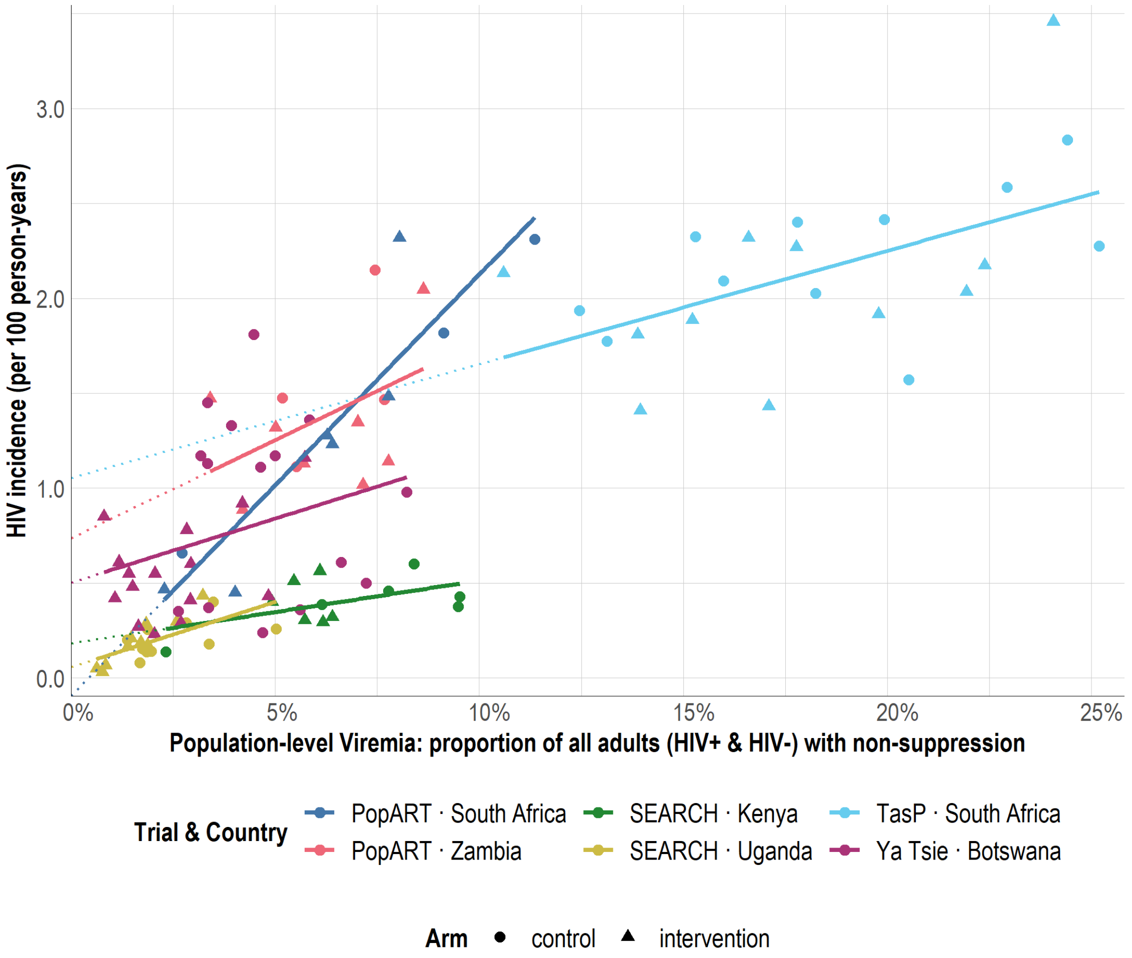

Table S7. Linear relationship between population-level viremia and HIV incidence, by trial and country

```
mod_s7 <- lm(incidence ~ trial_country:viremia + trial_country - 1, data = clusters)

mod_s7 %>%
  tbl_regression(
    estimate_fun = label_number(.0001),
    pvalue_fun = purrr::partial(style_pvalue, digits = 3)
  )
```

Characteristic	Beta	95% CI	p-value
Trial & Country			
PopART · South Africa	-0.0010	-0.0064, 0.0044	0.724
PopART · Zambia	0.0074	-0.0005, 0.0152	0.065
SEARCH · Kenya	0.0018	-0.0048, 0.0084	0.589
SEARCH · Uganda	0.0006	-0.0027, 0.0038	0.731

Characteristic	Beta	95% CI	p-value
TasP · South Africa	0.0105	0.0042, 0.0169	0.001
Ya Tsie · Botswana	0.0050	0.0024, 0.0076	<0.001
Trial & Country * Population-level viremia			
PopART · South Africa * Population-level viremia	0.2221	0.1455, 0.2987	<0.001
PopART · Zambia * Population-level viremia	0.1035	-0.0185, 0.2256	0.095
SEARCH · Kenya * Population-level viremia	0.0332	-0.0639, 0.1304	0.499
SEARCH · Uganda * Population-level viremia	0.0688	-0.0715, 0.2091	0.333
TasP · South Africa * Population-level viremia	0.0599	0.0257, 0.0940	<0.001
Ya Tsie · Botswana * Population-level viremia	0.0675	0.0039, 0.1310	0.038

Figure 3. Cross-gendered relationship between population-level viremia and HIV incidence, by trial.

```
cross_fm <-
  lm_plot(
    x = "viremia_female", y = "incidence_male",
    colour = "study", colour_label = "Trial",
    data = clusters %>% filter(!is.na(viremia_female))
  ) +
  xlab("Population-level Viremia (women)") +
  ylab("HIV incidence (men)") +
  expand_limits(y = 0.048, x = 0.29)

cross_mf <-
  lm_plot(
    x = "viremia_male", y = "incidence_female",
    colour = "study", colour_label = "Trial",
    data = clusters %>% filter(!is.na(viremia_male))
  ) +
  xlab("Population-level Viremia (men)") +
  ylab("HIV incidence (women)") +
  expand_limits(y = 0.048, x = 0.29)

up <- plot_grid(
  cross_mf + theme(legend.position = "none"),
  cross_fm + theme(legend.position = "none"),
  labels = c("a", "b", ""),
  label_size = 11,
  ncol = 2
)

plot_grid(
  up,
  cross_mf %>% get_legend(),
  ncol = 1,
  rel_heights = c(5, 1)
)
```

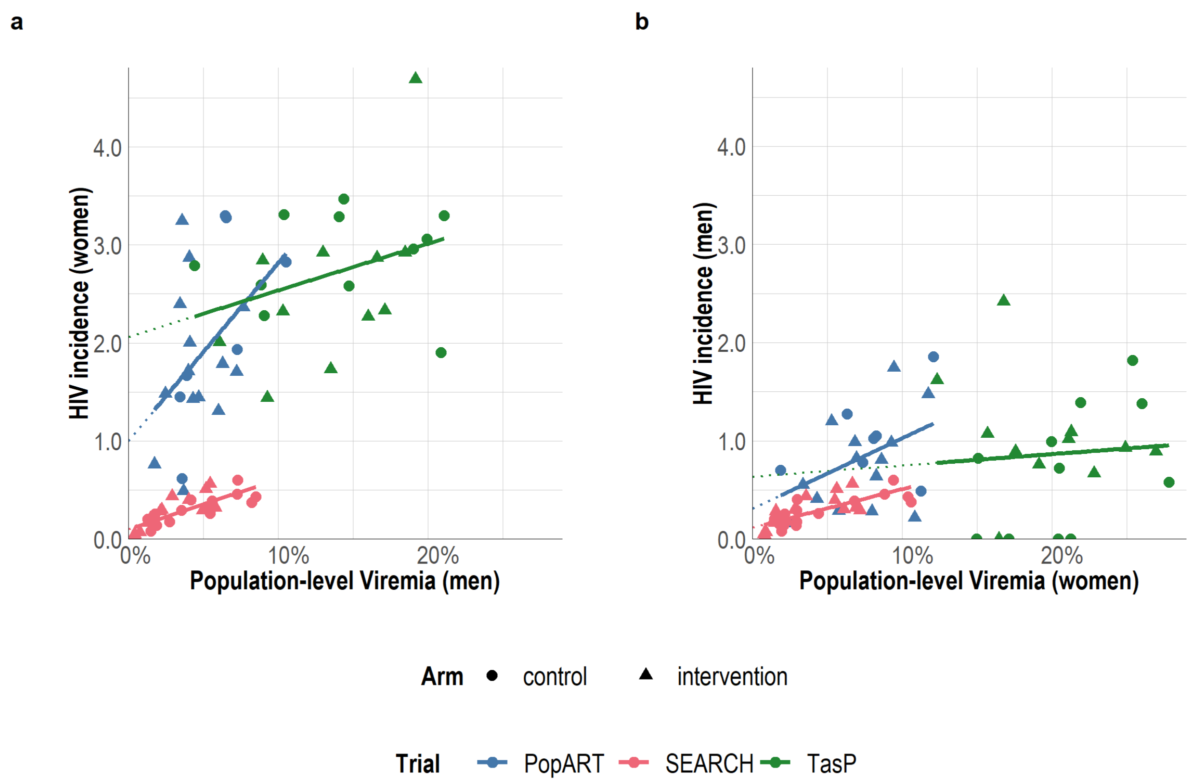

Table 4. Linear relationship between the prevalence of non-suppression (among PLHIV) and HIV incidence, by trial.

Summary of the approach

```
# Step 1
mod1 <- lm(incidence ~ viremia + study:prevalence + study - 1, data = clusters)

# Step 2
new_data <- clusters %>%
  expand(
    nesting(id, study, prevalence),
    non_suppression = seq(from = .05, to = .65, by = .03)
  ) %>%
  mutate(viremia = prevalence * non_suppression)
new_data$predicted_incidence <- predict(mod1, newdata = new_data)

# Step 3
mod2 <- lm(predicted_incidence ~ study:non_suppression + study - 1, data = new_data)
```

Bootstrap version to compute confidence intervals and p-values:

```
iteration_coef_mod2 <- function(data) {
  # a subsample with replacement
  # (sampling stratified by study)
  data <- data %>%
    dplyr::group_by(study) %>%
    sample_frac(replace = TRUE)
  mod1 <- lm(incidence ~ viremia + study:prevalence + study - 1, data = data)
```

```r
  new_data <- data %>%
    ungroup() %>%
    expand(
      nesting(id, study, prevalence),
      non_suppression = seq(from = .05, to = .65, by = .03)
    ) %>%
    mutate(viremia = prevalence * non_suppression)
    new_data$predicted_incidence <- predict(mod1, newdata = new_data)
    mod2 <- lm(predicted_incidence ~ study:non_suppression + study - 1, data = new_data)
    coef(mod2)
}

set.seed(2021) # for replicability

boot_coef <- lapply(1:1000, function(i){iteration_coef_mod2(clusters)}) %>%
  simplify2array %>%
  t()

boot_ci <- matrixStats::colQuantiles(boot_coef, probs = c(0.025, 0.975))
boot_sd <- matrixStats::colSds(boot_coef)

# compute p-values
beta <- coef(mod2)
zval <- beta / boot_sd
boot_p <- 2 * pt(-abs(beta / boot_sd), df = mod2$df.residual)

res <- cbind(coef = beta, boot_ci, p = boot_p)
res <- res %>% as_tibble(rownames = "term")
res$ci <- paste0(
  style_number(res$`2.5%`, digits = 4),
  ", ",
  style_number(res$`97.5%`, digits = 4)
)
```

```r
res %>%
  select(term, coef, ci, p) %>%
  gt::gt() %>%
  gt::fmt_number(2, decimals = 4) %>%
  gt::fmt(4, fns = purrr::partial(style_pvalue, digits = 3))
```

term	coef	ci	p
studyPopART	0.0099	0.0058, 0.0134	<0.001
studySEARCH	0.0006	-0.0017, 0.0024	0.574
studyTasP	0.0109	0.0003, 0.0196	0.022
studyYa Tsie	0.0054	0.0034, 0.0074	<0.001
studyPopART:non_suppression	0.0117	0.0020, 0.0241	0.032
studySEARCH:non_suppression	0.0056	0.0009, 0.0114	0.031
studyTasP:non_suppression	0.0170	0.0028, 0.0348	0.033
studyYa Tsie:non_suppression	0.0158	0.0025, 0.0327	0.033

Expected counterfactual incidence extraploated to scenario where 95-95-95 were achieved

```
compute_95_95_95 <- function(model) {
  marginaleffects::predictions(
    model,
    newdata = datagrid(non_suppression = 1 - .95^3, study = unique)
  ) %>%
    as_tibble() %>%
    select(study, incidence_95_95_95 = estimate)
}

iteration_95_95_95 <- function(i) {
  # a subsample with replacement
  # (sampling stratified by study)
  data <- clusters %>%
    dplyr::group_by(study) %>%
    sample_frac(replace = TRUE)
  mod1 <- lm(incidence ~ viremia + study:prevalence + study - 1, data = data)
  new_data <- data %>%
    ungroup() %>%
    expand(
      nesting(id, study, prevalence),
      non_suppression = seq(from = .05, to = .65, by = .03)
    ) %>%
    mutate(viremia = prevalence * non_suppression)
    new_data$predicted_incidence <- predict(mod1, newdata = new_data)
    mod2 <- lm(predicted_incidence ~ study:non_suppression + study - 1, data = new_data)
    compute_95_95_95(mod2) %>%
      mutate(iteration = i)
}

set.seed(2021) # for replicability

boot_data_95_95_95 <-
  1:1000 %>%
  map_dfr(iteration_95_95_95)
```

```
boot_ci_95_95_95 <-
  boot_data_95_95_95 %>%
  group_by(study) %>%
  summarise(
    incidence_95_95_95_low = quantile(incidence_95_95_95, probs = .025),
    incidence_95_95_95_high = quantile(incidence_95_95_95, probs = .975),
    .groups = "drop"
  )

res <- compute_95_95_95(mod2) %>%
  left_join(boot_ci_95_95_95, by = "study")

res %>%
  gt::gt() %>%
  gt::fmt_number(decimals = 4)
```

study	incidence_95_95_95	incidence_95_95_95_low	incidence_95_95_95_high
PopART	0.0116	0.0089	0.0140
SEARCH	0.0014	−0.0001	0.0025
TasP	0.0133	0.0052	0.0200
Ya Tsie	0.0076	0.0063	0.0093

Figure 4. Relationship between prevalence of non-suppression and HIV incidence, by trial

```
predict_data <- clusters
predict_data$incidence <- predict(mod2, newdata = clusters)

predict_data2 <- clusters %>%
  group_by(study) %>%
  summarise(non_suppression = range(0, non_suppression))
predict_data2$incidence <- predict(mod2, newdata = predict_data2)


lm_plot(x = "non_suppression", y = "incidence", colour = "study", colour_label = "Trial", lm = FALSE) +
  xlab("Prevalence of non-suppression: proportion of PLHIV with detectable viremia") +
  ylab("HIV incidence (per 100 person-years)") +
  geom_line(data = predict_data2, size = .5, linetype = "dotted") +
  geom_line(data = predict_data, size = 1)
```

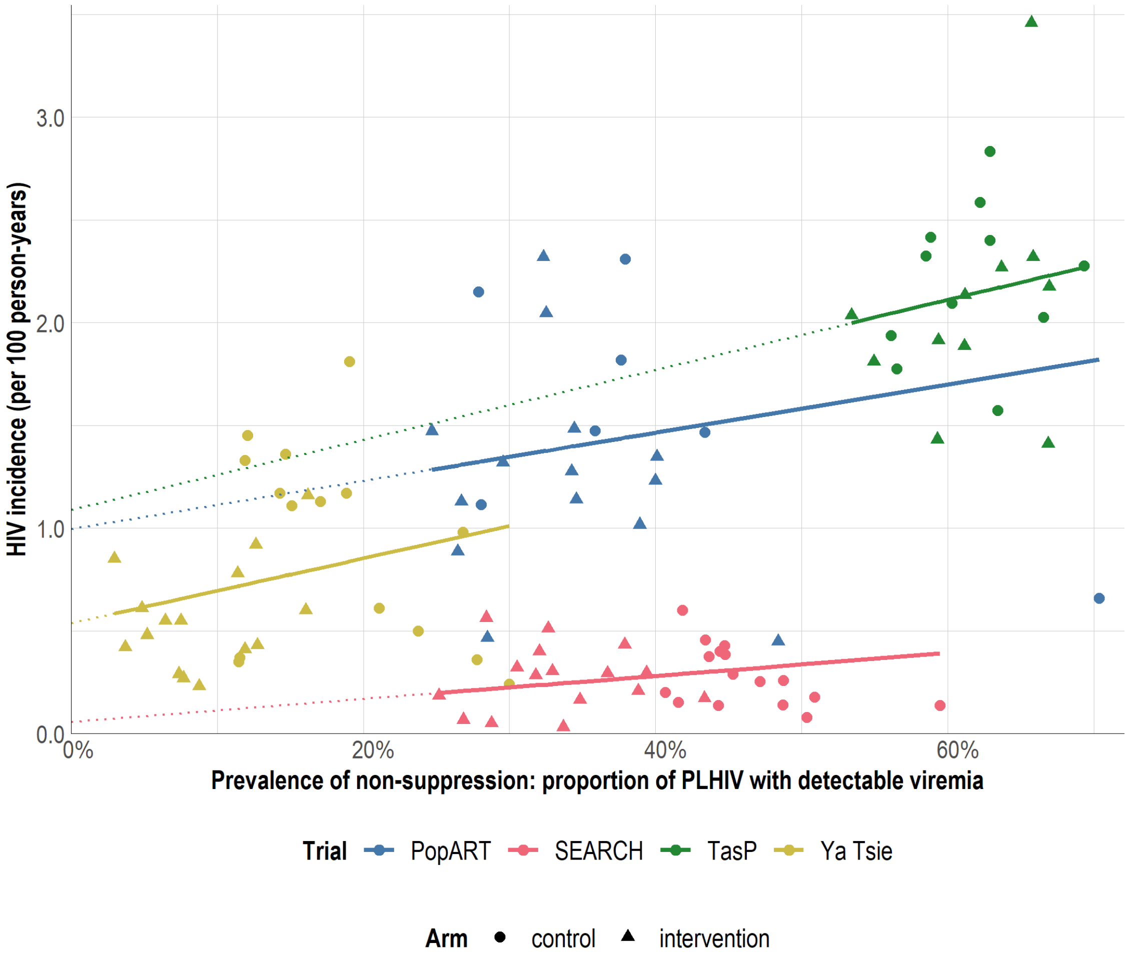

Table 5. Estimates of the magnitude of expected incidence reduction due to the observed reduction of viral suppression

```
non_supression_data <- tribble(
  ~study, ~arm, ~time_point, ~non_suppression,
  "PopART", "A", "baseline", .46,
  "PopART", "A", "endline", .31,
  "PopART", "B", "baseline", .45,
  "PopART", "B", "endline", .31,
  "PopART", "C", "baseline", .49,
  "PopART", "C", "endline", .40,
  "SEARCH", "C", "baseline", .59,
  "SEARCH", "C", "endline", .32,
  "SEARCH", "I", "baseline", .58,
  "SEARCH", "I", "endline", .21,
  "TasP", "C", "baseline", .74,
  "TasP", "C", "endline", .55,
  "TasP", "I", "baseline", .77,
```

```r
  "TasP", "I", "endline", .54,
  "Ya Tsie", "C", "baseline", .28,
  "Ya Tsie", "C", "endline", .17,
  "Ya Tsie", "I", "baseline", .30,
  "Ya Tsie", "I", "endline", .12,
)

compute_incidence_reduction <- function(model) {
  trends <- non_supression_data
  trends$pred_incidence <- predict(model, newdata = trends)
  trends %>%
    pivot_wider(names_from = "time_point", values_from = c(non_suppression, pred_incidence)) %>%
    mutate(
      incidence_reduction = pred_incidence_endline - pred_incidence_baseline,
      relative_reduction = abs(incidence_reduction) / pred_incidence_baseline
    )
}

compute_incidence_reduction(mod2) %>%
  select(study, arm, incidence_reduction, relative_reduction) %>%
  mutate(
    incidence_reduction = scales::percent(incidence_reduction, accuracy = .01, suffix = ""),
    relative_reduction = scales::percent(relative_reduction, accuracy = .1)
  ) %>%
  gt::gt()
```

study	arm	incidence_reduction	relative_reduction
PopART	A	-0.18	11.5%
PopART	B	-0.16	10.8%
PopART	C	-0.11	6.7%
SEARCH	C	-0.15	39.0%
SEARCH	I	-0.21	54.3%
TasP	C	-0.32	13.8%
TasP	I	-0.39	16.3%
Ya Tsie	C	-0.17	17.7%
Ya Tsie	I	-0.28	28.1%

Bootstrap version to compute 95% confidence intervals:

```r
iteration_incidence_reduction <- function(i) {
  # a subsample with replacement
  # (sampling stratified by study)
  data <- clusters %>%
    dplyr::group_by(study) %>%
    sample_frac(replace = TRUE)
  mod1 <- lm(incidence ~ viremia + study:prevalence + study - 1, data = data)
  new_data <- data %>%
    ungroup() %>%
    expand(
      nesting(id, study, prevalence),
      non_suppression = seq(from = .05, to = .65, by = .03)
    ) %>%
    mutate(viremia = prevalence * non_suppression)
```

```
    new_data$predicted_incidence <- predict(mod1, newdata = new_data)
    mod2 <- lm(predicted_incidence ~ study:non_suppression + study - 1, data = new_data)
    compute_incidence_reduction(mod2) %>%
      mutate(iteration = i)
}

set.seed(2021) # for replicability

boot_data <-
  1:1000 %>%
  map_dfr(iteration_incidence_reduction)
```

```
boot_ci <-
  boot_data %>%
  group_by(study, arm) %>%
  summarise(
    incidence_reduction_low = quantile(incidence_reduction, probs = .025),
    incidence_reduction_high = quantile(incidence_reduction, probs = .975),
    relative_reduction_low = quantile(relative_reduction, probs = .025),
    relative_reduction_high = quantile(relative_reduction, probs = .975),
    .groups = "drop"
  )

res <- compute_incidence_reduction(mod2) %>%
  left_join(boot_ci, by = c("study", "arm"))

res %>%
  mutate(
    incidence_reduction = paste0(
      scales::percent(incidence_reduction, accuracy = .01, suffix = ""),
      " [",
      scales::percent(incidence_reduction_low, accuracy = .01, suffix = ""),
      ", ",
      scales::percent(incidence_reduction_high, accuracy = .01, suffix = ""),
      "]"
    ),
    relative_reduction = paste0(
      scales::percent(relative_reduction, accuracy = .1),
      " [",
      scales::percent(relative_reduction_low, accuracy = .1, suffix = ""),
      ", ",
      scales::percent(relative_reduction_high, accuracy = .1, suffix = ""),
      "]"
    )
  ) %>%
  select(study, arm, incidence_reduction, relative_reduction) %>%
  gt::gt()
```

study	arm	incidence_reduction	relative_reduction
PopART	A	-0.18 [-0.36, -0.03]	11.5% [2.2, 21.2]
PopART	B	-0.16 [-0.34, -0.03]	10.8% [2.1, 20.1]
PopART	C	-0.11 [-0.22, -0.02]	6.7% [1.3, 12.2]
SEARCH	C	-0.15 [-0.31, -0.02]	39.0% [8.5, 61.9]

SEARCH	I	-0.21 [-0.42, -0.03]	54.3% [11.7, 86.9]
TasP	C	-0.32 [-0.66, -0.05]	13.8% [2.5, 25.3]
TasP	I	-0.39 [-0.80, -0.06]	16.3% [3.1, 29.5]
Ya Tsie	C	-0.17 [-0.36, -0.03]	17.7% [3.8, 27.8]
Ya Tsie	I	-0.28 [-0.59, -0.05]	28.1% [6.1, 43.2]
