# Supplementary material for: Population-level viremia predicts HIV incidence at the community level across the Universal Testing and Treatment Trials in eastern and southern Africa
Source: PLOS Glob Public Health. 2023 Jul 14;3(7):e0002157. doi: 10.1371/journal.pgph.0002157 (PMC10348573; doi:10.1371/journal.pgph.0002157)
Supplement: Attachment — Submitted filename: Rebuttal_letter_v2023-05-03.docx [file pgph.0002157.s008.docx]

Population-level viremia predicts HIV incidence at the community level across the Universal Testing and Treatment Trials in eastern and southern Africa

# Reviewer #1:

This article presents an analysis combining the data from 4 trials of UTT in different countries that came to similar but not entirely identical findings and pools the data to explore the relationship between population viremia and HIV incidence (among other relationships). The methods are strong and most of the limitations are discussed. The results are not at all surprising in terms of the form of the relationship, but with analyses like these the value is in the actual size of the effects. Below are specific comments about the work.

I am strongly supportive of what the authors have done.

Thank you for you support.

1. However, I do think that, as they note, the effects of any UTT approach is going to be population specific and as such, I think there needs to be some more discussion of this issue. There is a nice explanation of the potential for residual confounding but here I’m more concerned about effect modification. What are the factors that are likely to explain different effect sizes. Can the authors speculate in the discussion?

Thank you for this question. Interestingly, much of the heterogeneity in the relationship between viremia and incidence across studies, was no longer apparent when the relationship between non-suppression and incidence was evaluated. This suggests that much of what appeared to be effect modification was in fact due to differences in confounding structures across the studies. Specifically, a closer relationship between prevalence and incidence in POPART would explain the steeper slope. We now elaborate on this point in the discussion. We have also added additional discussion of factors likely to result in different effect sizes.

The corresponding paragraph in the Discussion now stands as:

“Despite these challenges, the estimated relationship between the prevalence of non-suppression among PLHIV and HIV incidence was fairly consistent across trials, suggesting that much of the heterogeneity in the observed association between population level viremia and incidence (i.e. apparent “effect modification”) across trials may simply be due to different confounding patterns (i.e., differences in the extent of unmeasured shared determinants of HIV prevalence and incidence). The magnitude of the effect of the prevalence of non-suppression among PLHIV on HIV incidence is likely influenced by contextual factors, however, including mobility, network characteristics and risk behaviours, and, particularly going forward, coverage of biomedical prevention such as pre-exposure prophylaxis.”

2. In the methods, I found it a bit hard to follow what time periods each data corresponded to and how many observations each cluster could contribute. Could a cluster contribute one measure of prevalence of suppression or two? And I assume they could only contribute one measure of incidence? If that’s correct, how do the two measures of viremia relate to the one measure of incidence? Of course, if I’ve misunderstood a brief sentence in the methods could clarify that.

For clarity, we added the following sentence in the Methods section: “The unit of analysis was trial clusters: each cluster contributed one observation point, with one measure of viremia and one measure of incidence. The clusters were unweighted in the analysis.”

3. For the baseline HIV prevalence and non-suppression numbers, can you provide confidence intervals so we can see how much uncertainty there is in each community?

Statistically speaking, TasP implemented an exhaustive population cohort and, as such, it would not be relevant to compute confidence intervals. SEARCH also implemented an exhaustive population cohort, but calculated confidence intervals accounting for the uncertainty due to persons not measured (a distinct source of uncertainty than sample size). In addition, all trials implemented distinct approaches to population-level estimates (cf. Table 2) making difficult the computation of such intervals for some trials and complicating their interpretation/ comparability. To provide a sense of uncertainty in each community, we have thus instead added in Table S3 the average number of observations per cluster used for each indicator.

4. In reading through the methods, my first thought was, why just a linear function, why not something more curved. Reviewing the data in the figures suggests that maybe a linear model is the right one, but I can’t be sure. Did you try something like a quadratic? If not, it would be worth checking it doesn’t add to the fit of the model. And either way, it is worth explaining in the methods why you chose a linear function.

Theoretically, the relationship between viremia and incidence would be expected to be proportional under the assumption of random mixing. Additionally, a linear model provides easily interpretable coefficients. We did explore a quadratic relationship: it added complexity but did not improve the model fit (residuals were not significantly reduced). Given the added difficulty of interpretation, we have elected not to include these results. We now state this explicitly:

It is now stated in the manuscript: “As a sensitivity analysis, we also explored a quadratic relationship; as it complicated interpretation without improving fit, results are not reported here.”

5. The results for PopART are so different from the rest, it really needs some explaining. There isn’t really any discussion of this in the discussion section. What explains this much larger effect size in one trial vs another. As Ive noted above, I think there could be lots of explanations that should be explored, but these don’t come up in the discussion.

Thank you; please see the response to the first point above. Specifically, the estimated relationship between non-suppression and incidence (which is not confounded by shared drivers of prevalence and incidence) was 0.12 in PpopART, 0.16 in TasP, 0.17 in Ya Tsie, and 0.06 in SEARCH. This suggests that what appeared to be a much larger “effect” in POPART was likely largely driven by differences in confounding of the prevalence/incidence relationship. SEARCH did appear to have a smaller effect. As we note, this may have been due to chance, but mobility may also have played a role, as noted in the Discussion.

6. Given the differences in testing protocols across studies it might help to see an analysis strategy by whether the control arms got universal testing.

Two trials implemented universal testing in their control arm (SEARCH and TasP) while the two others (PopART and Ya Tsie) did not. As the analysis was stratified by trial, it would be possible to compare these two groups. However, interestingly (see also responses to points 1 and 5), the slope of the line between non-suppression and incidence was similar across the trials, including those with and without universal testing.

7. It would also be helpful to see the results stratified by intervention and control arm to see if the effect of the intervention changed the relationship.

We agree. We have now added in supplementary materials a sensitivity analysis by trial and by trial arm (Figure S4 and Table S5). This sensitivity analysis is also now mentioned in the methods, results, and discussion.

Methods: “Additional sensitivity analyses included incorporating: 1) trial-arm specific slopes and intercepts; and, 2) trial and country specific slopes and intercepts.”

Results: “Sensitivity analyses further stratified by both trial and arm (Figure S4, Table S5) or both trial and country (Figure S6, Table S7) yielded similar estimates; all estimated slopes remained positive, with the exception of the control arm in Ya Tsie (-0.370, -1.434 to 0.694).”

Discussion: “Results were robust to sensitivity analyses, with the exception of the control arm in Ya Tsie, in which a non-significant negative relationship was observed; this may have been due to imprecision resulting from the small number of clusters; the smaller number of observations available to estimate incidence and viremia in this trial may also have contributed.”

8. I don’t know if this is possible, partly because of the timing of the data, but given the different trials found somewhat different results whether you could look at change in the exposures in relation to changes in the outcome. Perhaps change from baseline to midpoint in population viremia and change in incidence from baseline to endpoint?

Unfortunately, only SEARCH trial was designed to be able to estimate incidence at the beginning of the trial (year 1) and at the end (year 3), and only in the intervention arm. We report the reduction in incidence over time observed in the intervention arm in SEARCH in the discussion. “the trial showed that annual HIV incidence in the intervention arm during the trial decreased by 32% [95% confidence interval: 16% to 44%] (15), from 0.43 cases per 100 person-years between years 0 and 1 to 0.31 between years 2 and 3.”

For PopART, the control arm of SEARCH, TasP and Ya Tsie, data are not available to estimate the evolution of HIV incidence over time. This is why the current analysis was performed at mid-point. The expected incidence reduction due to the evolution of non-suppression between baseline and end-line was therefore estimated using the model adjusted at the mid-point (table 5).

9. I could just be misunderstanding, but is Table 1 missing the prevalence of unsuppressed?

Prevalence of non-suppression among PLHIV at baseline has been added to table 1.

10. In figures 2-4 it would help for each symbol’s size to represent the size of the cluster to give some sense for how precisely each of these values is likely to be measured.

Clusters were not weighted by population size in the analysis. We now state explicitly in the methods: “The clusters were unweighted in the analysis.” Because of this, we feel that making symbol size proportional to population size could mislead the reader about the performed analysis. Further, the number of persons used to estimate each measure varied across measures, and depending on each study’s design and data collected was often different from the cluster population size. Instead, we have now added the average population size of each cluster, as well as the number of persons used to estimate each measure, per trial, to table S3.

# Reviewer #2:

Many thanks for the opportunity to review this manuscript.

This is a well written, well conceptualized manuscript that uses novel methods to estimate the magnitude of the impact of changes in viral suppression among people living with HIV (and the community-level viremia) on HIV incidence. Here are some suggestions for authors to consider:

**Introduction**

1. Line 116 – It is not immediately clear what the authors mean by “cross gendered analysis”. I suggest making this explicit in the introduction rather than further down, as it is now.

It is now stated in the introduction: “We aimed to describe the direction and strength of the relationship between population-level viremia (the proportion of an entire community, irrespective of HIV status, with non-suppressed plasma HIV RNA) and HIV incidence and to assess how this relationship changed when performing a cross-gendered analysis (assuming that transmission is mainly through heterosexual sex), by looking at the relationship between men’s population-level viremia and women’s HIV incidence, and between women’s population-level viremia and men’s HIV incidence.”

**Methods**

2. Line 166 - the authors statement of “community-level” linear regression was unclear to me at first. After looking at their R code (thank you for providing it!), they use study-level, aggregate viremia as an independent variable and study as the fixed-effect. If so, this could be better phrased as aggregate (or ecological, study level…) analysis, rather than community (N = 105 clusters/communities, as stated in line 126) level. In short, I would clarify the language by stating that that HIV incidence was regressed on aggregate study-level viremia, or something along these lines.

We clarify that sentence. It now stands as: “The relationship between population-level viremia and HIV incidence was estimated, using data aggregated at the community level from both intervention and control arms, through a linear regression allowing for trial-specific slopes and intercepts to account for variations in the trials’ context, design, and measures. The unit of analysis was trial clusters: each cluster contributed to one observation point, with one measure of viremia and one measure of incidence. The clusters were unweighted in the analysis.”

3. Line 173 – in the analysis for the association between prevalence of non-suppression among PLHIV and HIV incidence, prevalence is measured at year two or endline in 3 out of 4 trials. Meanwhile incidence is measured at different points in time between 0-36 months. Therefore temporality between the two, especially given the authors’ use of causal methods (and sometimes language) is questionable. The authors briefly address this issue with one sentence in limitations, but I think it deserves further discussion/acknowledgement throughout the methods and discussion.

Thank you we agree. First, to be more specific, the prevalence of non-suppression was measured at midpoint for PopART, TasP and Ya Tsie and was an average of all available measures for SEARCH (table 2). For HIV prevalence, we used midpoint for TasP, an average for SEARCH and baseline estimates for PopART and Ya Tsie, assuming that HIV prevalence would be stable over time during the course of the trials. This is stated explicitly in Table 2. We agree completely that this is a limitation (arguably the biggest one) in any attempt to interpret the results causally. We have now replaced causal with associational language in several places where it had crept in. We also now state the dependence of any causal interpretation on time-ordering assumptions more explicitly in the methods.

“Under the causal assumptions in figure 1 (including time-ordering) and if the regression models are correctly specified, this approach estimates, for each trial, the average causal effect of a one-unit change in the prevalence of non-suppression among PLHIV on HIV incidence. As these assumptions are unlikely to hold (particularly given the complex time-dependence between prevalence, non-suppression and incidence), this analysis is best interpreted as a summary of the relationship between the prevalence of non-suppression in PLHIV and HIV incidence.”

We have also expanded the discussion of this limitation.

“Interpretation of the observed associations is further complicated by the lack of clear time-ordering between exposures and outcomes; non-suppression among PLHIV during the study may have affected HIV incidence, while incident HIV infections in turn may have contributed to non-suppression; our results thus provide a summary of the association between these two complex time-dependent processes.”

4. Line 204 and 205 – the authors refer to causal assumptions in the DAG and to their estimate as the “average causal effect of …”. I would be careful using this and other causal language, even though they are using a G-methods to conduct the analysis for the following reasons, which I should be addressed in the paper (discussion or limitations):

o The estimates are calculated at an aggregate/ecological/study level not individual, which is a barrier to making any causal statements.

o One of identifiability conditions for causality is consistency (the observed outcome for every treated individual equals her outcome if she had received treatment) and requires a well-defined, consistent intervention. As the authors pointed out throughout the paper the trial interventions varied widely, further questioning the interpretation of estimates as causal.

o If the HIV incidence in the community is high, we are also more likely to see more unsuppressed individuals in the community (higher viremia), due to the time it takes for linkage to care to take place further questioning the directionality of the estimates.

Thank you for this comment. We agree re the need for avoiding causal language, with the exception of discussion of our motivation for the use of these methods. In particular, we agree completely regarding the limitation imposed by time ordering, and now make the reviewers point explicitly in the discussion. Please see also response to comment 3 above.

For the record, we respectfully disagree regarding the inability to make causal statements at an aggregate level (certainly, causal relationships at an aggregate level cannot be used to infer corresponding relationships at an individual level, but they nevertheless may be causal and identifiable), as well as the need for a separate consistency assumption (in a Structural Causal Model framework as we use here consistency is an axiom not a distinct assumption) and the corresponding ability to make causal statements about treatments with inherent variability. There is a long and fascinating academic debate on each of these topics; however, given that we fundamentally agree with the reviewer about the need for great care on any causal claims here, and our corresponding edits to reflect this, we suggest that this is a moot discussion as it pertains to the current manuscript.

5. Line 208 – authors should clarify “community-based bootstrap” do they mean that “study” is their resampling unit when doing the bootstrap analysis?

Thank you. We now clarify that the cluster was the resampling unit: “a bootstrap approach, in which each cluster was sampled with replacement, was applied to compute 95% confidence intervals and p-values (19).”

6. Line 214 – I suggest using “associated with” or “predictive of” instead of “due to”.

The wording has been updated throughout the manuscript.

**Results**

7. Figure 1 – As the authors present it, the adjustment of the Viremia --> HIV incidence relationship for HIV prevalence (the collider) opens non-causal paths between E and O.

Figure 1 encodes the causal assumptions needed to identify the causal effect of E (non-suppression) on O (incidence). If prevalence affected E directly, then it is true that conditioning on prevalence would open up a non-causal path between E and O. However, in this DAG it does not (which is plausible in this case). Of note, in this DAG trial alone is sufficient to satisfy the backdoor criteria for the effect of E on O; however, prevalence is included in the adjustment set because, as a strong driver of incidence, adjustment for prevalence is expected to increase precision (and indeed this was verified). Figure 1 also makes clear that indeed the Viremia 🡪 HIV incidence relationship is confounded, even after adjustment for trial. It is likely that this confounding is driving the apparently larger “effect size” of viremia on incidence (see also response to Reviewer 1 above).

8. Would authors consider adjustment for some of the “beyond-trial” drivers of HIV acquisition e.g. HIV prevalence outside of the trial? This is especially relevant since external HIV acquisition could still occur outside of the community, given the cluster randomized nature of the studies.

Thank you. We agree about the role of external HIV acquisition. The most likely role of such factors, in our assessment, is as 1) effect modifiers – i.e. external mixing and the external environment would modify the true causal slope here (acknowledging as discussed by the reviewer and our responses above, that this true causal slope is unlikely to be identified); and 2) as one form of the shared drivers of prevalence and incidence noted in Figure 1, which motivate us to evaluate the non-suppression incidence relationship. We now make point 1) explicitly in the discussion (see also response to Reviewer one point 1). While it would be interesting to examine this empirically, comparable contemporaneous data to do so are not readily available, and we do not feel it is key to our results or their interpretation.

9. Table 3 and 4 – minor point, but some values are rounded at 2 while others at 3 decimal points. Best to stick with one for consistency.

We were using a significant digits rule (1 digit over 0.20, two digits below 0.20, three digits below 0.05). It has been harmonized to now present systematically three digits.

10. Thank you for providing your code and data for replication – always helpful for students!

Thank you for your comment.

**Discussion**

11. Line 303 – Authors hypothesize that HIV infection in men could be driven by infections acquired outside of the community (again in 308/309). Further, trials are often at risk of selection bias, such that people (communities in this case) enrolled are not representative of the HIV transmission patterns in the general population. Given this, have the authors considered adjusting for HIV prevalence outside of the trial community to account for any residual confounding due to infections acquired outside of the trial “boundaries?”

Please see response to point 8 above.

12. Line 307 – this is a minor point, but have the authors considered reporting on the 95% VLS scenario instead of 100% to better align with the UNAIDS goals?

It has been added to Table 4.

Methods: “We also reported, using step 3 model, that would be the expected incidence per trial if UNAIDS 95-95-95 objectives were reached.”

Results: “Extrapolating to scenario where UNAIDS 95-95-95 objectives were reached (corresponding to a prevalence of non-suppression equal to 14.3%) resulted in an estimated HIV incidence of 1.16 [0.89 to 1.40] per 100 person-years in PopART, 0.14 [ 0.01 to 0.25] in SEARCH, 1.33 [0.52 to 2.00] in TasP, and 0.76 [0.63 to 0.93] in Ya Tsie.”

# Reviewer #3:

This study by Joseph Larmarange and colleagues describes the relationship between the prevalence of unsuppressed HIV viraemia and HIV incidence, as well as the relationship between the proportion of PLHIV who are unsuppressed and HIV incidence, at a population level. As might be expected, and in line with previous studies, both relationships are strongly positive, although it is interesting to see heterogeneity in the extent of the positive relationship. The study is based on data from four cluster-randomized controlled trials that evaluated the effectiveness of ART as a population-level intervention to reduce HIV incidence (“treatment as prevention”). This is a large and important dataset, which adds considerable weight to the argument that reductions in HIV viraemia can be expected to reduce HIV incidence – despite the four trials being somewhat inconsistent in their findings about the impact of treatment as prevention. The study is well-written and clear.

Thank you.

**Minor comments**

1. The assumption of a linear relationship between HIV incidence and the level of viraemia could be questioned, particularly in the presence of significant heterogeneity in risk. (Key populations such as PWID, FSW and MSM might contribute disproportionately to transmission even though they only account for a small fraction of the population.) Instead of fitting a model of the form y = a + bx, where y is the incidence rate and x is the prevalence of unsuppressed viraemia, one might fit a model of the form y = a + x^b, or even just y = x^b. The authors do a good job of explaining why it’s appropriate to include the intercept term (a) in the context of a cRCT, but taken out of context (for example in a mathematical model of the impact of treatment as prevention at a national level), it could be troublesome. I suspect that fitting these alternative regression models would do little to change the overall conclusions of the paper, and I don’t feel strongly that they should be included, but I include this point as an observation, which the authors can ignore if they choose.

As answered to reviewer 1, we did explore a quadratic relationship: it added complexity but did not improve the model (residuals were not significantly reduced). We now state this explicitly in the methods “As a sensitivity analysis, we also explored a quadratic relationship; as it complicated interpretation without improving fit ,results are not reported here.”

2. Abstract, line 122: “increase in suppression” rather than “reduction of non-suppression”.

The sentence has been updated.

3. Table 1: It’s not clear what the (12-15) in the row headings at the bottom represents. Maybe these are citations, though it’s not clear they’re necessary.

It was the source of already published data. It is now mentioned in the note of the table.

4. In Figure 2, PopART looks like an outlier, in terms of the steepness of the slope. What would explain this? Maybe there is heterogeneity between Western Cape and Zambia? Did the authors consider fitting separate lines for the Western Cape and Zambian sites?

A sensitivity analysis by study and country has been added (cf. figure S6 and table S7); we found broadly consistent results. This is now described in the methods and noted in the results and discussion.

5. Last paragraph of p. 14: Another explanation for the difference between male and female slopes (which is slightly different from the point about differences in transmission probabilities) is that female incidence is higher than male incidence, so you’d expect the slope to be greater (in absolute terms).

The corresponding sentence in the discussion has been expanded to reflect that point: “This finding could reflect that the probability of HIV transmission from women to men is lower than from men to women, resulting in lower male incidence than female incidence and, therefore, lower slopes.”

6. In the same paragraph, (line 304) “also found” suggests the results are consistent, but they actually seem to be inconsistent (?). If the Rakai study found greater male reductions in HIV incidence, would that not suggest a greater slope in men?

Thank you. We agree. We have cut “also” to make this interesting observation accurate.

7. A general grammatical issue: The authors tend to talk about “an increase of HIV incidence” (or other metrics) when it would be more conventional to say “an increase in HIV incidence”. Similarly for “a reduction of…”. One would say “an increase of 5%” but not “an increase in 5%”.

The wording has been updated through the manuscript.

# Reviewer #4:

I enjoyed reading this paper and found it well conceived and explained. I have only a few minor comments:

Thank you.

Line 248- “HIV prevalence heterogeneous…”

Here I think it would be more helpful to give the ranges across all the trials combined instead of across the whole sample as that would better support the point you are making. eg. the within-trial range in HIV prevalence was between 20 and 30 percentage points etc.

Thank you. We agree this is important information. In the results we provide the ranges across all the trials combined as follows:

“Communities were also heterogenous (table S3). Across all trials, community-level HIV prevalence, measured in 257 929 persons, varied from 2.2% to 41.1% between communities; community-level prevalence of non-suppression among PLHIV, measured in 31 377 persons, from 3.0% to 70.4%; community-population-level viremia from 0.6% to 25.2%; and community-level HIV incidence, measured over 345 844 person-years, from 0.03 to 3.46 per 100 person-years.”

Line 294 “importance of incorporating metrics of non-suppression among PLHIV when predicting incidence,” . Should that be population viremia, since that paragraph is referring to viremia and the following paragraph is about non-suppression.

Thank you. We have modified the sentence to read: “These findings are consistent with prior findings that population-level viremia (which takes into account both the prevalence of non-suppression and HIV prevalence) is a strong predictor of HIV incidence (20), and the importance of incorporating metrics of that take into account non-suppression among PLHIV when predicting incidence, especially since the scale-up of antiretroviral treatment.”

Line 156- Population-level viremia, also known as the prevalence of detectable virus and defined as the prevalence of non-suppressed HIV infections among all resident adults, irrespective of HIV status, was obtained by multiplying the prevalence of non-suppression (among PLHIV) at midpoint and HIV prevalence.

This definition doesn’t quite match with the data given in the supplementary material i.e. the viremia estimate isn’t the product of prevalence and non-supression but perhaps just a rounding difference but would be worth noting.

Thank you for this question. Viremia was calculated for each cluster separately as the product of cluster-specific prevalence and cluster-specific non-suppression. In table S3, we are presenting medians and not means. The median cluster for HIV prevalence is not the same cluster for the median suppression.

# Reviewer #5:

Thank you for the opportunity to review this comprehensive, informative analysis on population viremia and HIV incidence in the four UTT trials in eastern and southern Africa. I recall seeing this work presented in conference, and I’m very pleased to finally see it summarized for publication. The work is novel, thorough, and provides critical epidemiological insights. The manuscript was well written, analysis well done, and the tables and figures are clear and to the point. I have only minor comments.

Thank you.

1) How were biases in study participation dealt with in these analyses? How sensitive would results be to different assumptions about differential participation in the survey by HIV and viremia status? Could selection biases into the incidence and viremia cohorts potentially explain to some extent why achieving 100% suppression in PLHIV would not achieve 100% reductions in HIV incidence?

Thank you for this excellent question. As each study was different, each study employed distinct approaches to adjustment for potential bias due to differential study participation. These approaches are detailed in several trial-specific publications, with additional cross-trial comparisons below:

Sabapathy K, Balzer L, Larmarange J, Block L, Floyd S, Iwuji C, Wirth K, Ayles H, Fidler S, Kamya M, Petersen M, Havlir D, Dabis F, Moore J, Hayes R. Achieving the UNAIDS 90-90-90 targets: a comparative analysis of four large community randomised trials delivering universal testing and treatment to reduce HIV transmission in sub-Saharan Africa. BMC Public Health. 2022 Dec 13;22(1):2333. doi: 10.1186/s12889-022-14713-5. PMID: 36514036; PMCID: PMC9746009.

Balzer LB, Ayieko J, Kwarisiima D, Chamie G, Charlebois ED, Schwab J, van der Laan MJ, Kamya MR, Havlir DV, Petersen ML. Far from MCAR: Obtaining Population-level Estimates of HIV Viral Suppression. Epidemiology. 2020 Sep;31(5):620-627. doi: 10.1097/EDE.0000000000001215. Erratum in: Epidemiology. 2021 Sep 1;32(5):e25. PMID: 32452912; PMCID: PMC8105880.

We agree; however, that residual bias in these estimates is possible, and that this might contribute to the non-zero intercept and now note this.

“Extrapolation of our results to a hypothetical scenario in which 100% of PLHIV were suppressed predicted that residual HIV infections would still occur. As suggested by phylogenetic analysis (23), some HIV seroconversions are likely driven by population mobility and HIV acquisition from outside communities. This finding suggests that improving ART coverage and viral suppression in isolated communities, as occurred in the UTT trial designs, might not be sufficient to stop HIV transmission; challenges in both the time-ordering and estimation of population-level measures may also have contributed.”

2) It would be helpful if the authors referred to community level prevalence estimates accordingly throughout the manuscript. There are times when the authors are referring to overall prevalence estimates in the cohorts and then to community level estimates. Even in the abstract I found it confusing.

Thank you. We now clarify this in the abstract. In the main text we now clarify when we are discussing community-specific measures as follows:

“Communities were also heterogenous (table S3). Across all trials, community-level HIV prevalence, measured in 257 929 persons, varied from 2.2% to 41.1% across communities; community-level prevalence of non-suppression among PLHIV, measured in 31 377 persons, from 3.0% to 70.4%; community-population-level viremia from 0.6% to 25.2%; and community-level HIV incidence, measured over 345 844 person-years, from 0.03 to 3.46 per 100 person-years.”

3) Why were the VL suppression cutoffs chosen for the various cohorts? Were these the lower limit of detection of the assays used?

We followed the definition of viral suppression used by each trial according to their respective protocols. It was usually based on the detection limit of the used assays and/or on national policies.

4) Could the authors please expand briefly upon how community bootstrapping was done?

Please see answer to comment 5 from reviewer 2.

5) The authors hypothesize that the stronger relationship between male viremia and female HIV incidence vs. female viremia and male HIV incidence could be due to either lower probability of male infection or higher probability of external HIV acquisition among men. Is it possible to adjust for community male circumcision prevalence in these trials, or was prevalence too low? The authors mention external male infection from outside the community, but observations could also be due to unsampled key populations or possibly higher male contact rates. It might be worth expanding here. Is it also possible the weaker correlation with male incidence might be because we are under sampling male HIV incident cases?

This is an intriguing suggestion, but it is unfortunately not feasible for us to include community male circumcision. Regarding unsampled key populations, please see response to point 1 above.

6) The author recommend surveillance for population viremia, but don’t specify exactly how that should best be done. What are their recommendations?

This is a topic of high importance but requires more nuance than can be accommodated in the current manuscript (particularly in light of the reviewers points 1 and 5 above). We now state in the discussion:

“however, generating accurate population level estimates requires care to account for non-representative participation (26,27).”
