# Supplementary material for: Population-level viremia predicts HIV incidence at the community level across the Universal Testing and Treatment Trials in eastern and southern Africa
Source: PLOS Glob Public Health. 2023 Jul 14;3(7):e0002157. doi: 10.1371/journal.pgph.0002157 (PMC10348573; doi:10.1371/journal.pgph.0002157)
Supplement: Attachment — Submitted filename: Rebuttal_letter_v2023-06-19.docx [file pgph.0002157.s009.docx]

Population-level viremia predicts HIV incidence at the community level across the Universal Testing and Treatment Trials in eastern and southern Africa

# Editor

The main point that we would like further considered is further discussion explaining the results around heterogeneity, particularly the important explanation "This suggests that what appeared to be a much larger “effect” in POPART was likely largely driven by differences in confounding of the prevalence/incidence relationship." would be helpful to elaborate in the Discussion to communicate the causal inference results to a wider non-specialist audience (and see also further comments from Reviewer #1).

Thank you. Please see our response to point 1, reviewer 1, below.

I tend to agree with the reviewer regarding the interpretation of confidence intervals from a local census.

Thank you. Please see our response to point 2, reviewer 1, below.

# Reviewer #1:

Overall I'm happy with the changes and commend the authors on the revisions, but there are two points that I think are worth coming back to.

Thank you very much.

1. First, the issue of heterogeneity. It seems very unlikely that the effects of changes in population level viremia on incidence wouldn't be population specific. As such, the note that much of the effect disappears when accounting for prevalence seems not sufficient. I'd like to see this discussed more, not just listed as a limitation as it doesn't seem like your data is sufficient to answer the question definitively and so more comment on this seems important.

Thank you. We now do not discuss this as a limitation, but include a paragraph dedicated to the discussion of this nuanced finding, include additional elaboration on potential sources of heterogeneity. Specifically, in the Discussion:

“Interestingly, the estimated relationship between the prevalence of non-suppression among PLHIV and HIV incidence was surprisingly consistent across trials, suggesting that one major contributor to the heterogeneity in the observed association between population level viremia and incidence (i.e. apparent “effect modification”) across trials may have been the presence of different confounding patterns (i.e., differences in the extent of unmeasured shared determinants of HIV prevalence and incidence) across the studies. Nonetheless, the effect of local population-level viremia on HIV incidence is likely to vary across settings, as a result of differences in factors such as including mobility, sexual network characteristics and risk behaviors, and, particularly going forward, coverage of biomedical prevention such as pre-exposure prophylaxis.”

2. Second, you note that you don't need confidence intervals on the prevalence because this is a population census. I disagree with this approach and take the view expressed in Modern Epidemiology that all populations, even if a census, can be seen as a sample of a larger super population. I wouldn't go to the mat over this and leave it to the editor to decide if this an important enough change, but it does seem worth noting.

Following the reviewer’s comment, we completed table 1, by adding 95% confidence intervals for baseline HIV prevalence and baseline prevalence of non-suppression, taking into account the clustered design of each trial, and following the different adjustments described in table 2. Table 5 has also been updated accordingly.

# Reviewer #2:

The authors have appropriately addressed my comments regarding the paper. Thank you taking the time.

Thank you very much/
